# Near-infrared-driven photothermal atom transfer radical polymerization

**DOI:** 10.1039/d5sc07153d

**Published:** 2025-11-27

**Authors:** Martyna Cybularczyk-Cecotka, Filip Bandalewicz, Wiktor Lewandowski, Grzegorz Szczepaniak

**Affiliations:** a Faculty of Chemistry, University of Warsaw Pasteura 1 02-093 Warsaw Poland g.szczepaniak@uw.edu.pl

## Abstract

Reversible-deactivation radical polymerization (RDRP) is a powerful tool in modern polymer chemistry, enabling the synthesis of well-defined materials with complex architectures. Among RDRP methods, photoinduced atom transfer radical polymerization (photo-ATRP) and photoinduced reversible addition–fragmentation chain transfer (photo-RAFT) polymerization are two of the most common approaches. However, in the context of biological applications, their use is often hampered by their reliance on UV light and their sensitivity to oxygen. Herein, we present a photothermal approach utilizing gold nanobipyramids (NBPs) to drive both ATRP and RAFT polymerizations in aqueous media under aerobic conditions. By precisely tuning the morphology of NBPs, we harnessed their ability to generate localized heating upon near-infrared (NIR) light irradiation (780 nm). This localized heating efficiently triggered radical generation from a water-soluble azo initiator (2,2′-azobis(2-amidinopropane)dihydrochloride, AAPH). The resulting radical flux enabled well-controlled ATRP of oligo(ethylene oxide) methyl ether methacrylate (OEOMA_500_) at low volume (250 µL) in a 96-well plate open to air. The photothermal ATRP exhibited excellent temporal control, enabling rapid on/off switching of polymerization simply by NIR light modulation. The versatility of our methodology was further demonstrated by its successful application in photo-RAFT polymerization, achieving controlled polymerization of various monomer classes under aqueous conditions. This robust, nanotechnology-enabled photothermal approach opens new avenues for advanced materials synthesis and high-throughput applications by overcoming key limitations of traditional photo-RDRP systems.

## Abstract 2

Gold nanobipyramids efficiently convert NIR light into heat to drive ATRP and RAFT polymerization in water, eliminating the need for deoxygenation.

## Graphical abstract

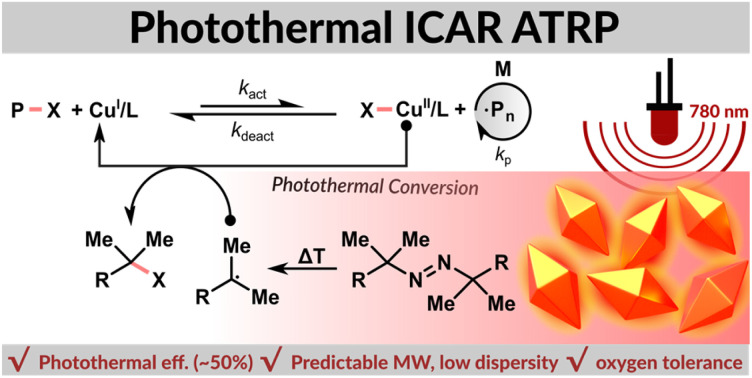

## Introduction

Precise control over polymer synthesis is paramount in the development of advanced materials. Reversible-deactivation radical polymerization (RDRP) has emerged as a cornerstone technique, offering unprecedented control over molecular weight distribution and polymer architecture.^1–3^ Among the RDRP toolbox, photoinduced atom transfer radical polymerization (photo-ATRP) and photoinduced reversible addition–fragmentation chain transfer (photo-RAFT) polymerization provide exceptional spatiotemporal control.^4–6^ However, these methods typically require UV light irradiation,^7–9^ which is biocidal and limits their applications. Shifting to near-infrared (NIR) light overcomes this critical barrier, offering deeper tissue penetration, enhanced scalability, and superior biocompatibility,^10,11^ making it ideal for biomedical applications.^12^

Despite the advantages of using NIR light, its insufficient photon energy often precludes the direct generation of radicals. While upconversion is a widely explored strategy to address this,^13–17^ its practical feasibility is often limited by the need for high-power irradiation, which raises concerns regarding potential damage to biological materials. The alternative approach uses photocatalysts that efficiently absorb NIR light and subsequently transfer electrons to molecules to generate radicals.^18–43^ However, these systems frequently rely on toxic transition metals or necessitate complex, multi-step synthetic procedures, hindering their widespread use. Therefore, novel approaches to NIR-driven RDRP are being sought.

Among the emerging strategies to render NIR light effective in an RDRP setting,^11^ photothermal conversion presents a particularly attractive route. This process involves the transduction of absorbed light energy into heat, leading to localized temperature increases that can drive chemical reactions.^44–52^ Gold nanoparticles (Au NPs) are exceptionally efficient photothermal transducers due to their unique localized surface plasmon resonance (LSPR) properties.^53,54^ This phenomenon involves the collective oscillation of conduction-band electrons. Importantly, at their resonance wavelength Au NPs exhibit plasmon-mediated absorption cross-sections that are orders of magnitude larger than those of molecular chromophores. This absorption rapidly heats the electron gas, which then rapidly transfers energy to the Au NP lattice, establishing a new thermal equilibrium and causing rapid lattice heating. Overall, this photo-to-thermal energy conversion occurs efficiently within picosecond timescales.

A key advantage of Au NPs is their highly tunable optical properties. By tailoring their particle shape, the LSPR resonance wavelength can be precisely tuned, covering the visible to NIR spectrum (520–1200 nm), with anisotropic particles being particularly effective for the longer wavelengths. This tunability, combined with their high photothermal conversion efficiency, straightforward surface functionalization, and biocompatibility, makes Au NPs ideal candidates for diagnostic and therapeutic applications.^55^

In polymer chemistry, these properties have been exploited to enable photoinduced radical generation.^56–58^ For example, Barner-Kowollik and colleagues demonstrated the use of gold nanorods as photothermal conversion agents to initiate free radical polymerization in an aqueous medium under NIR irradiation (800 nm).^57^ The localized thermal energy generated in this process induces the decomposition of a radical initiator, yielding primary radicals that subsequently initiate polymerization. While this photothermal approach presents a promising avenue for NIR-driven radical generation, integrating it with an RDRP technique is essential for achieving precise control over molecular weight distribution and polymer architecture.

ATRP is based on a reversible redox process that relies on the dynamic exchange of a halogen atom (X) between a dormant polymer chain end (C(sp^3^)–X) and a copper catalyst, specifically a [Cu^I^/L]^+^ activator.^3,59^ This equilibrium allows for controlled polymer growth by maintaining a low concentration of active radicals. However, molecular oxygen poses a significant challenge as it reacts with propagating radicals to form unreactive peroxy radicals, disrupting polymerization.^60^ Additionally, oxygen oxidizes the [Cu^I^/L]^+^ activator to [Cu^II^/L]^+^, effectively quenching the reaction.^61^ Consequently, conventional ATRP requires meticulous degassing to eliminate oxygen. To mitigate this sensitivity, oxygen-tolerant ATRP strategies have emerged.^60,62^ These approaches focus on regenerating the [Cu^I^/L]^+^ activator from the [X–Cu^II^/L]^+^ deactivator, essentially acting as an *in situ* oxygen scavenger.^63–66^ This regeneration can be triggered by diverse chemical,^67^ biological,^68,69^ or physical stimuli.^70–72^ A particularly effective approach is initiators for continuous activator regeneration (ICAR) ATRP.^3^ This method utilizes an external initiator to continuously regenerate the [Cu^I^/L]^+^ activator.^67^ However, traditional ICAR ATRP typically relies on bulk heating for radical generation, which can limit spatial and temporal control.

**Fig. 1:**
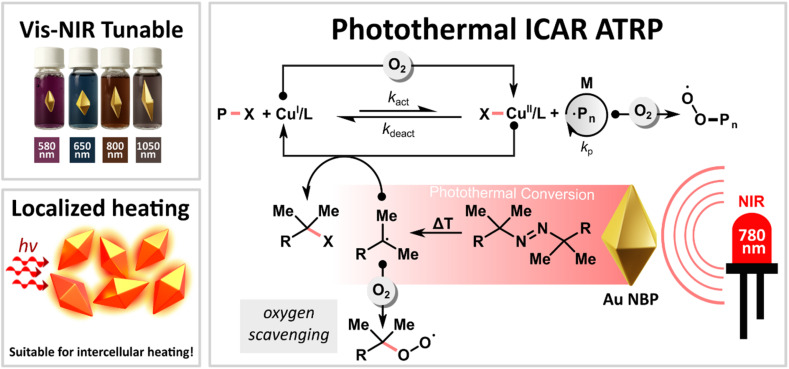
Proposed mechanism of NBPs mediated photothermal ICAR ATRP.

To overcome these limitations, we developed a novel oxygen-tolerant photothermal ICAR ATRP technique (Fig. 1), drawing inspiration from the previous work of Barner-Kowollik on NIR-induced free radical polymerization.^57^ Our method leverages anisotropic gold nanoparticles as photothermal conversion agents in conjunction with copper catalysis. By tuning the Au NPs' optical characteristics, colloidal stability, and reaction conditions, we achieved controlled polymerization in aqueous solutions open to air, using NIR light (780 nm). We also demonstrated the applicability of the developed approach to photo-RAFT polymerization. This work demonstrates how the unique light-matter interactions of nanomaterials can be harnessed to drive efficient and well-controlled RDRP in aqueous media.

## Results and discussion

### Au NPs design and synthesis

We selected gold nanobipyramids (NBPs) from various anisotropic nanostructures due to their tunable longitudinal LSPR, which allows for optimizing their absorption in the NIR region.^73–75^ This property, combined with their large absorption cross sections and strong electromagnetic field concentration at their sharp tips, results in superior photothermal conversion efficiency.^76–78^ Compared to the more traditionally utilized nanorods, NBPs exhibit a higher absorption-to-scattering ratio because of their sharper spectral features.^79,80^ This enhanced ratio directly improves light-to-heat conversion efficiency, making NBPs highly effective agents for photothermal applications.

**Fig. 2:**
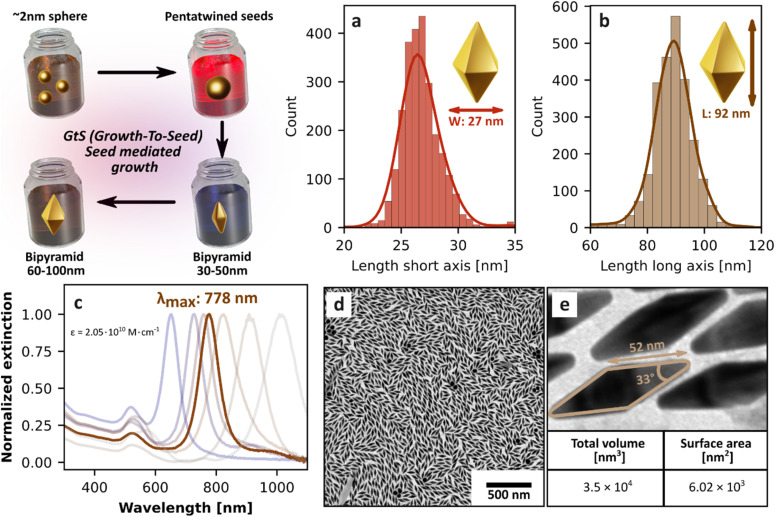
Schematic illustration of the seed-mediated growth protocol, showing morphological evolution from ∼2 nm spherical seeds to pentatwinned intermediates, and ultimately to small and large NBPs. (a and b) Size distribution histograms of NBP dimensions based on analysis of 2500 particles: (a) long axis (tip to tip length) and (b) short axis (width), showing narrow dispersion and consistent morphology. (c) UV-Vis-NIR absorption spectra of NBPs synthesized with varying AgNO_3_, HAuCl_4_ concentration (see Table S2), demonstrating plasmonic tunability and calculated extinction coefficient (*ε* = 2.05 × 10^10^ M·cm^−1^). The selected NBPs exhibit a longitudinal surface plasmon resonance (LSPR) peak at 778 nm (highlighted), with a full width at half maximum (FWHM) of ∼80 nm. (d and e) TEM micrographs of the final NBPs; (e) shows a magnified region of (d).

The NBPs used in our system were synthesized *via* a modified seed-mediated growth method, as previously described by Chateau *et al.* (Fig. 2 and S1).^81^ In this method, parameters such as silver ion concentration were adjusted to control the aspect ratio, while gold precursor concentration during the overgrowth stage was tuned to obtain different sizes (Table S1). Together, these parameters enabled precise tuning of the LSPR peak to match the NIR LED irradiation wavelength around 780 nm (Fig. 2c and Table S1). The obtained NBPs exhibited high shape purity (above 95%), narrow size distribution (long axis 91.6 ± 7.5 nm, short axis mean: 27.2 ± 3.4 nm), and narrow LSPR band set to 778 nm, which are characteristics convenient for NIR photothermal applications.

### Photothermal conversion

**Fig. 3:**
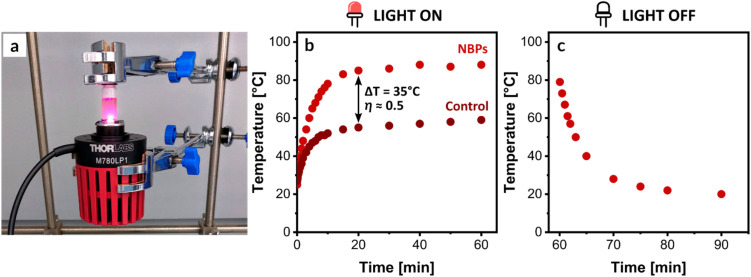
Photothermal performance of NBPs. (a) Illustration of the experimental setup for photothermal ICAR ATRP. (b) Temperature evolution curve of an aqueous NBP solution as a function of time. The solution was irradiated with a 780 nm NIR LED for 60 min, (c) after which the LED was turned off to allow for natural cooling.

The photothermal conversion efficiency (*η*) of NBPs was investigated by monitoring the bulk temperature change of the reaction solution under NIR irradiation (780 nm) (Fig. 3a). An aqueous solution of NBPs with a total concentration of Au^0^ = 188 µM (Absorbance_780_ = 0.383, path length = 2 mm), as calculated based on extinction measurements (see SI), exhibited a rapid increase in temperature when exposed to NIR irradiation, reaching a maximum of 87 °C after 10 min (Fig. 3b). This substantial temperature increase corresponds to high photothermal conversion efficiency (*η* ≈ 0.5, Table S2 and Fig. S5), confirming that synthesized NBPs are effective agents for photothermal RDRP.

It is critical to note that these measurements represent the average bulk solution temperature. Given the localized nature of plasmonic heating, substantial thermal gradients are anticipated in the immediate vicinity of individual nanoparticles, leading to significantly higher local temperatures at the nanoscale.^53^ Upon cessation of NIR irradiation, a rapid decrease in the bulk solution temperature was observed, indicative of efficient heat dissipation (Fig. 3c). A control experiment, conducted in the absence of NBPs, resulted in a maximal temperature of 52 °C after 10 min of NIR irradiation (Fig. 3b), confirming the substantial contribution of NBP-mediated photothermal conversion to the observed temperature increase.

### Polymerization conditions

High-throughput approaches accelerate materials discovery by enhancing efficiency, boosting productivity, and reducing reagent consumption and costs.^82,83^ While RDRP techniques have been increasingly integrated with high-throughput platforms for rapid reaction optimizations and polymer libraries generation,^83–88^ the necessity for external deoxygenation in small volumes presents a significant practical challenge,^89^ especially with volatile reagents. Oxygen-tolerant RDRP methodologies effectively circumvent this limitation, eliminating the need for degassing and substantially simplifying low-volume polymerization workflows.^26,60^

To demonstrate the performance of photothermal ICAR ATRP in a low-volume (250 µL), high-throughput manner, we performed polymerizations of oligo(ethylene oxide) methyl ether methacrylate (OEOMA_500_, *M*_*n*_ = 500) in an aqueous solution containing 4% (v/v) DMSO in a 96-well plate open to air (Table 1 and Fig. S6). The reactions were driven by NIR LED irradiation (780 nm, 0.9 W cm^−2^), with NBPs serving as the photothermal conversion agents. 2-Hydroxyethyl α-bromoisobutyrate (HOBiB) was used as the initiator, copper(ii) bromide (CuBr_2_) as the catalyst, and tris(2-pyridylmethyl)amine (TPMA) as the ligand (L). 2,2′-azobis(2-amidinopropane)dihydrochloride (AAPH), with a half-life (*t*_1/2_) of 10 h at 56 °C, was used as the external radical source. Furthermore, cetyltrimethylammonium chloride (CTAC) was added as a surfactant to both stabilize the NBPs and impede uncontrolled polymerization by suppressing the dissociation of the [X–Cu^II^/L]^+^ deactivator to the [Cu^II^/L]^2+^ complex and a free halide anion.^90^ CTAC concentration was set to 20 mM, which is above the critical micellization concentration (CMC) of CTAC in aqueous solution (∼1 mM) and below the threshold for surfactant-induced aggregation (typically >100 mM). To ensure reproducibility, all experiments were performed in triplicate (see Table S4), with the data in Table 1 representing the outcomes most representative of the triplicate average.

**Table 1:** Optimization of photothermal ICAR ATRP of OEOMA_500_^a^

Entry	[Au^0^] (µM)	[CuBr_2_] (equiv.)	[TPMA] (equiv.)	[AAPH] (equiv.)	Conv.^b^ (%)	*M*_*n*,th_ c	*M*_*n*,abs_ d	*M*_*n*,app_ e	e *Đ*	*I*_eff_ f (%)
1	375	0.2	0.4	1.0	91	91 200	56 800	47 000	1.37	161
2	188	0.2	0.4	1.0	92	92 200	58 400	48 100	1.29	158
3	94	0.2	0.4	1.0	92	92 200	63 000	51 200	1.26	146
4	94	0.2	0.4	0.5	73	73 200	60 200	49 600	1.23	122
5	94	0.2	0.4	0.25	62	62 200	57 700	47 600	1.22	108
6	94	0.2	0.4	0.19	58	58 200	57 800	47 600	1.26	100
7	94	0.3	0.6	0.19	50	50 200	51 800	43 500	1.23	97
8	188	0.3	0.6	0.19	60	60 200	59 200	48 600	1.21	102
9^g^	94	0.3	0.6	0.19	45	45 200	46 500	39 800	1.24	97
10^h^	94	0.3	0.6	0.19	0	—	—	—	—	—

a Reaction conditions: [OEOMA_500_]/[HOBiB]/[CuBr_2_]/[TPMA]/[AAPH] = 200/1/*x*/*x*/*x*, [OEOMA_500_] = 300 mM, [Au^0^] = 94–375 µM, [CTAC] = 20 mM. Polymerizations were conducted in an aqueous solution containing DMSO (4% v/v) at a scale of 250 µL in an open-air 96-well plate. NIR LED irradiation (780 nm, 0.9 W cm^−2^) was applied for 30 min.

b Determined by ^1^H NMR spectroscopy.

c *M*_*n*,th_ = 200 × conv. × MW_OEOMA500_ + MW_HOBIB_.

d Determined by Mark–Houwink calibration.^63^

e Determined by SEC analysis (DMF as eluent) calibrated with poly(methyl methacrylate) standards.

f Initiation efficiency (*I*_eff_) = *M*_*n*,th_/*M*_*n*,abs_.

g VA-044 instead of AAPH.

h VA-086 instead of AAPH, with 1.5 h of NIR irradiation.

Photothermal ICAR ATRP was initially investigated using molar ratios of [OEOMA_500_]/[HOBiB]/[CuBr_2_]/[TPMA]/[AAPH] = 200/1/0.2/0.4/1 and [Au^0^] = 375 µM (Table 1, entry 1). After 30 min of NIR irradiation, ^1^H NMR analysis showed 91% conversion of OEOMA_500_. However, size exclusion chromatography (SEC) revealed a relatively broad molecular weight distribution (*Đ* = 1.37) and a significant discrepancy between the experimentally determined number-average molecular weight (*M*_*n*,abs_ = 56 800) and the theoretical value (*M*_*n*,th_ = 91 200, Table 1, entry 1). Reducing the NBP loading by two- and four-fold (to 188 µM and 94 µM of [Au^0^], respectively) resulted in improved polymerization control without sacrificing monomer conversion (Table 1, entries 2 and 3). We attribute this to a slower decomposition of AAPH.

Varying the AAPH initiator concentration had a pronounced effect on polymerization performance (Table 1, entries 3–6). Decreasing the AAPH concentration from 1.5 mM to 0.28 mM resulted in a well-controlled polymerization (*Đ* = 1.26) with a predetermined molecular weight (*M*_*n*,th_ = 58 200, *M*_*n*,abs_ = 57 800) and 58% monomer conversion (Table 1, entry 6). In ICAR ATRP under open-to-air conditions, radicals generated from AAPH homolysis remove dissolved oxygen and react with [X–Cu^II^/L]^+^, regenerating the [Cu^I^/L]^+^ activator. However, excessive AAPH concentrations can disrupt polymerization control due to the generation of a high radical flux, leading to increased chain initiation and termination events. This likely explains the observed decrease in experimental molecular weights (*M*_*n*,abs_) compared to theoretical values (*M*_*n*,th_) at higher AAPH concentrations (Table 1, entries 3–5).

The effect of copper concentration was subsequently investigated, and the results indicated that increasing the [X–Cu^II^/L]^+^ deactivator concentration improved polymerization control, with dispersity values decreasing from 1.26 to 1.23 when the CuBr_2_ concentration was increased from 0.3 mM to 0.45 mM (Table 1, entry 7). However, this improvement in control was accompanied by a moderate monomer conversion (50%). Thus, we decided to test if increasing the NBP loading by two-fold with this optimized ratio of [CuBr_2_]/[TPMA]/[AAPH] = 0.3/0.6/0.19 could lead to better results; indeed, we achieved higher monomer conversion (60%) while further lowering dispersity to 1.21 (Table 1, entry 8).

Finally, the impact of water-soluble azo initiators with differing decomposition temperatures was evaluated. 2,2′-Azobis[2-(2-imidazolin-2-yl)propane]dihydrochloride (VA-044, *t*_1/2_ = 10 h at 44 °C) and 2,2′-azobis[2-methyl-*N*-(2-hydroxyethyl)propionamide] (VA-086, *t*_1/2_ = 10 h at 85 °C) were selected for this study. The use of VA-044 resulted in moderate monomer conversion of 45% (Table 1, entry 9). Conversely, VA-086 proved to be entirely inactive under the given reaction conditions, a consequence of its insufficient decomposition rate at the reaction mixture temperature (Table 1, entry 10).

Based on these systematic investigations, we confirmed that the proposed approach is feasible for open-air, high-throughput (96-well plate) systems, and the optimized conditions for the photothermal ICAR ATRP of OEOMA_500_ were determined to be [OEOMA_500_]/[HOBiB]/[CuBr_2_]/[TPMA]/[AAPH] = 200/1/0.3/0.6/0.19 (Table 1, entry 8).

### Control experiments

While initial polymerizations were conducted in open-air vessels to confirm the method's robustness, subsequent experiments were set up in sealed vials with a larger reaction volume (1 mL *vs.* 250 µL) to prevent solvent evaporation and demonstrate scalability (Table 2 and Fig. S7). Furthermore, to explore the synthesis of higher molecular weight polymers, the target degree of polymerization (DP_T_) was increased to 400. This was achieved by reducing the HOBiB initiator concentration by half to 0.75 mM, while maintaining the optimized concentrations of the other components. This adjustment combined with an extension of the reaction time to 1 h resulted in higher monomer conversion (72%) without significant changes in dispersity (*Đ* = 1.19) and molecular weight control (*M*_*n*,th_ = 144 200, *M*_*n*,abs_ = 136 000; Table 2, entry 1).

**Table 2:** Control experiments^a^

Entry	Deviation from standard conditions	Conv.^b^ (%)	*M*_*n*,th_ c	*M*_*n*,abs_ d	*M*_*n*,app_ e	*Đ* e	*I*_eff_ f (%)
1	—	72	144 200	136 000	96 600	1.19	106
2	No NBPs	0	—	—	—	—	—
3	No AAPH	0	—	—	—	—	—
4	No CTAC	84	168 200	133 200	95 000	1.75	126
5	No HOBiB	72	—	352 600	212 000	1.92	—
6	No irradiation, heating to 88 °C	61	122 200	119 300	86 700	1.26	102
7	NRs instead of NBPs	71	142 200	122 200	88 400	1.26	116
8	NSTs instead of NBPs	70	140 200	126 200	90 800	1.25	111

a Standard conditions: [OEOMA_500_]/[HOBiB]/[CuBr_2_]/[TPMA]/[AAPH] = 400/1/0.6/1.2/0.38, [OEOMA_500_] = 300 mM, [HOBiB] = 0.75 mM, [CuBr_2_] = 0.45 mM, [TPMA] = 0.9 mM, [AAPH] = 0.285 mM, [Au^0^] = 188 µM, [CTAC] = 20 mM. Polymerizations were conducted in an aqueous solutions containing DMSO (4% v/v) at a scale of 1 mL in sealed vials in the presence of air (non-degassed solutions). NIR LED irradiation (780 nm, 0.9 W cm^−2^) was applied for 60 min.

b Determined by ^1^H NMR spectroscopy.

c *M*_*n*,th_ = 400 × conv. × MW_OEOMA500_ + MW_HOBIB_.

d Determined by Mark–Houwink calibration.^63^

e Determined by SEC analysis (DMF as eluent) calibrated with poly(methyl methacrylate) standards.

f Initiation efficiency (*I*_eff_) = *M*_*n*,th_/*M*_*n*,abs_.

Notably, no polymerization was observed after 1 h of NIR irradiation when either gold nanoparticles (Table 2, entry 2) or AAPH (Table 2, entry 3) were excluded. In contrast, the absence of CTAC resulted in higher monomer conversion of 84%. However, this led to uncontrolled polymerization, as indicated by a broad molecular weight distribution (*Đ* = 1.75). This can be ascribed to the known issue in aqueous Cu-catalyzed ATRP where the [X–Cu^II^/L]^+^ deactivator undergoes significant dissociation into the [Cu^II^/L]^2+^ complex and a free halide anion (X^−^).^90,91^ The resultant ‘naked’ [Cu^II^/L]^2+^ complex is unable to function as an effective deactivator, thereby compromising polymerization control. The addition of CTAC, which contains a chloride anion, not only stabilizes the NBPs but also suppresses the dissociation of the [X–Cu^II^/L]^+^ deactivator. As anticipated, the exclusion of the HOBiB initiator yielded a polymer characterized by a significantly higher molecular weight (*M*_*n*,abs_ = 352 600) and a high dispersity (*Đ* = 1.92) (Table 2, entry 5). Replacing NIR irradiation with bulk heating in the presence of NBPs resulted in decreased monomer conversion (61%) and increased dispersity (*Đ* = 1.26) (Table 2, entry 6). Finally, substituting the NBPs with other Au NP morphologies, specifically nanorods (NRs) and nanostars (NSTs) (Table 2, entries 7 and 8, Fig. S9), yielded comparable monomer conversion but compromised polymerization control (Table S5).

These control experiments collectively underscored the critical role of the NBPs in achieving well-controlled photothermal ICAR ATRP under NIR irradiation.

### Colloidal stability of Au NPs

To evaluate the impact of polymerization conditions on the stability and optical properties of the nanoparticles, UV-Vis-NIR spectra of the reaction mixtures were recorded before and after ATRP.

For NRs and NSTs (Fig. S8a and b), polymerization induced significant band broadening and red-shifting, indicative of interparticle coupling and aggregation. These spectral changes were accompanied by macroscopic color fading, *i.e.*, a transition from deep brown (NRs) and blue (NSTs) to lighter, less saturated hues. In contrast, NBPs (Fig. S8c) exhibited only minor spectral changes, with a slight blue-shift of the LSPR maximum to ∼738 nm, consistent with partial tip rounding.

Based on the absorbance near 400 nm, both NRs and NBPs showed negligible loss of dispersed gold. NSTs, however, displayed a pronounced decrease in intensity, reflecting reduced colloidal stability and sedimentation of nanoparticle aggregates. Consequently, these findings identify NBPs as the most robust morphology for photothermal polymerization.

### Kinetic study

**Fig. 4:**
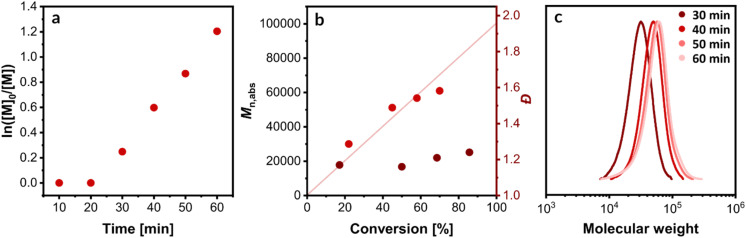
Polymerization of OEOMA_500_. (a) First-order kinetic plot of ln([*M*]_0_/[*M*]) *versus* time. (b) Evolution of number-average molecular weight (*M*_*n*_) and dispersity (*Đ*) with monomer conversion. (c) Evolution of SEC traces over time.

The photothermal ICAR ATRP of OEOMA_500_ was performed under optimized conditions ([OEOMA_500_]/[HOBiB]/[CuBr_2_]/[TPMA]/[AAPH] = 200/1/0.3/0.6/0.19) in the presence of NBPs. Aliquots were withdrawn at predetermined intervals and quenched with 1,4-bis(3-isocyanopropyl)piperazine to terminate polymerization.^92^ Samples were subsequently analyzed using ^1^H NMR and SEC (Fig. 4 and S10).

Kinetic analysis revealed an initial induction period of approximately 20 min, attributed to the consumption of dissolved oxygen by radicals generated from AAPH decomposition (Fig. 4a). Following this induction period, a rapid polymerization ensued, achieving 73% monomer conversion within 60 min from the reaction start. The observed molecular weights exhibited a linear increase with increasing monomer conversion, closely aligning with theoretical predictions, while maintaining low dispersity values (*Đ* < 1.24) (Fig. 4b). Furthermore, SEC chromatograms showed monomodal peaks that shifted towards higher molecular weights as polymerization progressed (Fig. 4c). Consistent polymerization kinetics were observed for a target degree of polymerization of 400 (Fig. S11 and S12). The kinetic profile of this photothermal.

ICAR ATRP aligns with typical free radical polymerization, demonstrating a rate dependence primarily on the external initiator decomposition rate, rather than the ATRP equilibrium constants.^3^

### Temporal control

**Fig. 5:**
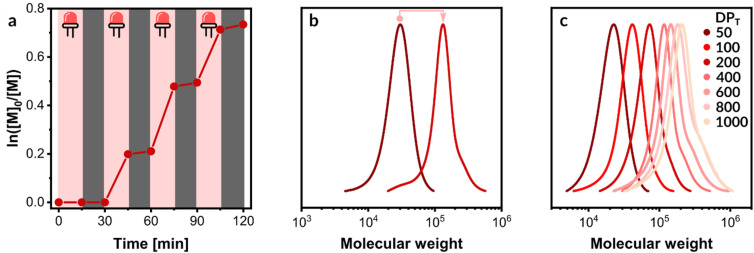
Photothermal ICAR ATRP. (a) Polymerization kinetics under intermittent “ON/OFF” light irradiation. (b) Chain extension from a macroinitiator to form a diblock copolymer. (c) A series of polymers synthesized with various targeted degrees of polymerization (DP_T_).

While conventional ICAR ATRP provides control over polymerization, its reliance on bulk thermal heating for radical generation presents a significant drawback in achieving fine temporal control. The inherent thermal inertia of bulk heating systems limits the ability to rapidly initiate and halt polymerization, as heat dissipation is slow.^46^ We hypothesized that employing photothermal conversion with NBPs and NIR irradiation would impart superior temporal control, enabling rapid on/off switching of the polymerization process. Indeed, following three cycles of on/off irradiation, a monomer conversion of approximately 60% was attained, demonstrating excellent temporal control over the polymerization (Fig. 5a).

To further evaluate the temporal control, we addressed a key concern: the presence of oxygen, which oxidizes the activator to [Cu^II^/L]^+^ complex, thereby halting polymerization. We conducted polymerization under anaerobic conditions to isolate the photothermal effect (Fig. S13). As anticipated, degassing the reaction mixture resulted in a faster polymerization rate. Crucially, even after rigorous deoxygenation, we observed only a slight increase in monomer conversion after the NIR irradiation was turned off. This provides strong evidence for the high degree of temporal control imparted by our photothermal system, confirming that the polymerization is effectively halted when the NIR light is turned off.

### Block copolymerization

Chain-end fidelity was confirmed through a chain extension experiment. A p(OEOMA_500_) macroinitiator (DP_T_ = 100, conv. = 39%, *M*_*n*,abs_ = 27 500, *Đ* = 1.19) was synthesized at a polymerization scale of 1 mL (Fig. 5b). This macroinitiator was directly used in a chain extension reaction with OEOMA_500_ (DP_T_ = 400). After 30 min of NIR irradiation, a monomer conversion of 47% was achieved. Subsequent SEC analysis revealed a clear shift to higher molecular weights (*M*_*n*,app_ = 109 900, *M*_*n*,abs_ = 159 100, *Đ* = 1.24) and the absence of low molecular weight shoulders, thereby confirming a high chain-end fidelity (Fig. 5b).

### Modulating the molecular weights

Achieving precise polymer synthesis with narrow molecular weight distributions across a broad molecular weight range while mitigating oxygen inhibition remains a significant challenge. Thus, the developed photothermal ICAR ATRP method was further evaluated for synthesizing polymers with DP_T_ up to 1000 (Table 3). This was achieved by varying the HOBiB concentration (1.5–0.3 mM) while maintaining constant concentrations of other polymerization components. Within 1 h, monomer conversions reached 69–74%, yielding polymers with low dispersities (*Đ* = 1.19–1.34). However, for target DPs of 800 and 1000, notable deviations from theoretical molecular weights and pronounced tailing in SEC traces were observed (Table 3, entries 6 and 7, Fig. 5c). This tailing indicates ongoing chain initiation by radicals generated from the photothermal homolytic cleavage of AAPH. At higher HOBiB concentrations (lower DP_T_), the contribution of radicals from AAPH decomposition to the overall chain initiation is negligible compared to the initiation from HOBiB. Conversely, as HOBiB concentration decreases (higher DP_T_), the impact of AAPH decomposition on the polymer molecular weight becomes more significant.

**Table 3:** Photothermal ICAR ATRP of OEOMA_500_ with varying DP_T_^a^

Entry	DP_T_	[HOBiB] (mM)	Conv.^b^ (%)	*M*_*n*,th_ c	*M*_*n*,abs_ d	*M*_*n*,app_ e	*Đ* e	*I*_eff_ f (%)
1	50	6.0	69	17 500	19 400	19 400	1.18	90
2	100	3.0	72	36 200	34 300	31 000	1.19	111
3	200	1.5	73	73 200	62 900	51 100	1.20	116
4	400	0.75	73	146 200	136 000	96 600	1.19	106
5	600	0.5	70	210 200	162 700	112 000	1.25	129
6	800	0.375	71	284 200	202 800	134 300	1.30	140
7	1000	0.3	74	370 200	242 400	155 600	1.34	153

a Reaction conditions: [OEOMA_500_] = 300 mM, [HOBiB] = 0.3–6.0 mM, [CuBr_2_] = 0.45 mM, [TPMA] = 0.9 mM, [AAPH] = 0.285 mM, [Au^0^] = 188 µM, [CTAC] = 20 mM. Polymerizations were conducted in an aqueous solutions containing DMSO (4% v/v) at a scale of 1 mL in sealed vials in the presence of air (non-degassed solutions). NIR LED irradiation (780 nm, 0.9 W cm^−2^) was applied for 60 min.

b Determined by ^1^H NMR spectroscopy.

c *M*_*n*,th_ = DP_T_ × conv. × MW_OEOMA500_ + MW_HOBIB_.

d Determined by Mark–Houwink calibration.^63^

e Determined by SEC analysis (DMF as eluent) calibrated with poly(methyl methacrylate) standards.

f Initiation efficiency (*I*_eff_) = *M*_*n*,th_/*M*_*n*,abs_.

### Photothermal RAFT polymerization

Finally, to further demonstrate the advantages of the proposed methodology, we investigated the utility of NBPs as photothermal conversion agents in photo-RAFT polymerization under NIR irradiation (Table 4 and Fig. S15). This approach demonstrated exceptional versatility, enabling successful controlled polymerization of three distinct classes of monomers: methacrylates, acrylates, and acrylamides (Table 4). Specifically, OEOMA_500_, 2-[2-(2-methoxyethoxy)ethoxy]ethyl acrylate (MEA), and *N*,*N*-dimethylacrylamide (DMA) were polymerized under aqueous conditions with high monomer conversions (68–98%) and low dispersity values (*Đ* < 1.28), confirming the broad applicability and universality of our technique.

**Table 4:** Photothermal RAFT polymerization^a^

Entry	Monomer	CTA	Time (min)	Conv.^b^ (%)	*M*_*n*,th_ c	*M*_*n*,app_ d	*Đ* d
1	OEOMA_500_	CPADB	60	68	68 300	58 700	1.28
2	MEA	DDMAT	60	73	32 200	28 400	1.23
3	DMA	DDMAT	30	98	19 800	21 400	1.24

a Reaction conditions: [monomer]/[CTA]/[AAPH] = 200/1/0.19, [monomer] = 300 mM, [CTAC] = 20 mM, [Au^0^] = 188 µM. Polymerizations were conducted in an aqueous solutions containing DMSO (4% v/v) at a scale of 1 mL in sealed vials in the presence of air (non-degassed solutions) under NIR LED irradiation (780 nm, 0.9 W cm^−2^). [CPADB] = 4-cyano-4-(phenylcarbonothioylthio)pentanoic acid, [DDMAT] = 2-(dodecylthiocarbonothioylthio)-2-methylpropanoic acid.

b Determined by ^1^H NMR spectroscopy.

c *M*_*n*,th_ = 200 × conv. × MW_monomer_ + MW_CTA_.

d Determined by SEC analysis (DMF as eluent) calibrated with poly(methyl methacrylate) standards.

## Conclusions

We have developed an oxygen-tolerant, photothermally driven ATRP system using gold nanobipyramids (NBPs) as photothermal agents. The synthesized NBPs exhibited an exceptional photothermal conversion efficiency (*η* ≈ 0.5), enabling a rapid and substantial temperature increase (Δ*T* = 35 °C) in aqueous solutions under 780 nm irradiation, which effectively drove the polymerization. We established robust polymerization conditions in a high-throughput, 96-well plate format open to air, eliminating the need for deoxygenation and simplifying workflows for low-volume (250 µL) polymerization.

Through systematic optimization of NBPs, external radical source (AAPH) and copper catalyst ([X–Cu^II^/L]^+^) concentrations, well-controlled polymerizations with predetermined molecular weights and low dispersity values (*Đ* = 1.19–1.34) were achieved. Control experiments unequivocally demonstrated the indispensable roles of NBPs and the AAPH in driving the photothermal ICAR ATRP. Kinetic studies confirmed a controlled polymerization process with a linear increase in molecular weight with monomer conversion and low dispersities. Furthermore, this photothermal approach provided superior temporal control over polymerization, allowing for rapid on/off switching of the reaction through NIR irradiation. The high chain-end fidelity of the synthesized polymers was confirmed through successful block copolymerization. We also demonstrated the versatility of this methodology for synthesizing polymers with a broad range of molecular weights (*M*_*n*,abs_ = 19 400–242 400).

Finally, the broad applicability of this photothermal strategy was extended to photoinduced RAFT polymerization, showcasing its versatility for the controlled polymerization of various monomer classes, including methacrylates, acrylates, and acrylamides, under aqueous conditions.

This research provides a significant advancement in oxygen-tolerant reversible-deactivation radical polymerization in aqueous media, opening new avenues for efficient and precise polymer synthesis.

## Author contributions

M. C. C: investigation; methodology; formal analysis; visualization; validation; writing – review and editing. F. B: investigation; methodology; formal analysis; visualization; validation; writing – review and editing. W. L: funding acquisition; methodology; project administration; resources; supervision; writing – review and editing. G. S: conceptualization; methodology; visualization; funding acquisition; project administration; resources; supervision; writing – original draft. All authors approved the final version of the manuscript.

## Conflicts of interest

There are no conflicts to declare.

## Supplementary Material

SC-OLF-D5SC07153D-s001

## Data Availability

The data that support the findings of this study are available in the supplementary information (SI). Supplementary information is available. See DOI: https://doi.org/10.1039/d5sc07153d.
