# Supplementary material for: Near-infrared-driven photothermal atom transfer radical polymerization
Source: Chem Sci. 2025 Nov 27;17(5):2654–63. doi: 10.1039/d5sc07153d (PMC12687368; doi:10.1039/d5sc07153d)
Supplement: SC-017-D5SC07153D-s001 [file SC-017-D5SC07153D-s001.pdf]

Supplementary Information (SI) for Chemical Science.
This journal is © The Royal Society of Chemistry 2025

Supplementary Information

Near-infrared-driven photothermal atom transfer radical polymerization

Martyna Cybularczyk-Cecotka, Filip Bandalewicz, Wiktor Lewandowski* and Grzegorz Szczepaniak*

Faculty of Chemistry, University of Warsaw, Pasteura 1, 02-093 Warsaw, Poland.

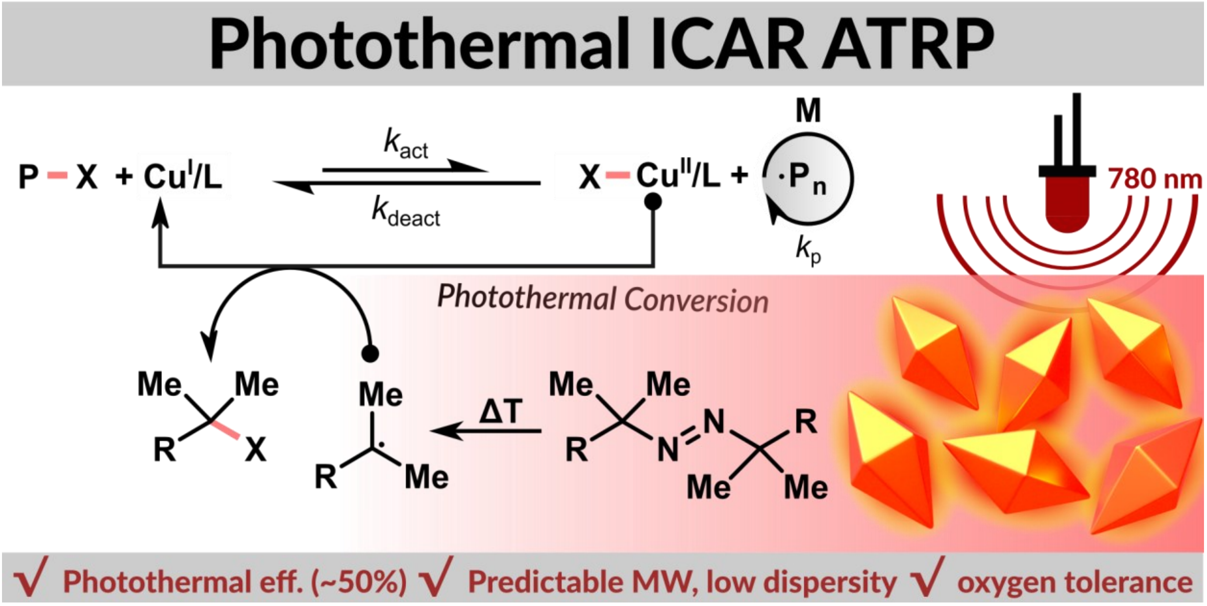

Supplementary Information

Table of contents

Experimental Details........4

Materials........4

Instrumentation........4

Nuclear Magnetic Resonance (NMR)........4

Size Exclusion Chromatography (SEC)........4

Transmission Electron Microscopy (TEM)........5

Ultraviolet–Visible-Near Infrared Spectrophotometry (UV-Vis-NIR)........5

Image Analysis........5

Temperature Measurements........5

Polymerizations........5

Procedures........6

Gold Nanobipyramids (NBPs) Synthesis (Figure 2)........6

Gold Nanorods (NRs) Synthesis.........7

Gold Nanostars (NST) Synthesis.........8

Photothermal Conversion Efficiency Measurements (Figure 3)........9

Heat-Transfer Analysis (Figure 3b)........11

Extinction Coefficient of NBPs (Figure 2c)........14

Optimization of Photothermal ICAR ATRP of $OEOMA_{500}$ (Table 1)........15

Control Experiments of Photothermal ICAR ATRP (Table 2)........18

Colloidal Stability of NPs........19

Impact of Nanoparticle Morphology on Photothermal ICAR ATRP.........20

Kinetics of Photothermal ICAR ATRP of $OEOMA_{500}$ (Figure 4)........22

Temporal Control of Photothermal ICAR ATRP (Figure 5a)........23

Temporal Control of Photothermal ICAR ATRP Under Anaerobic Conditions........24

Block Copolymerization (Figure 5b)........24

Synthesis of p($OEOMA_{500}$)-*b*-p($OEOMA_{500}$) Diblock Copolymer........24

Synthesis of p($OEOMA_{500}$)-*b*-p($OEOMA_{276}$) Diblock Copolymer........25

Supplementary Information

Photothermal ICAR ATRP of $OEOMA_{500}$ with Varying DP_T (Table 3) 26

Photothermal RAFT Polymerization of Different Monomers (Table 4) 26

Supplementary Information

Experimental Details

Materials

All chemicals were purchased from commercial vendors and, unless otherwise noted, used without further purification. Tris(2-pyridylmethyl)amine (**TPMA**, >98%) and 2-hydroxyethyl 2-bromoisobutyrate (**HOBiB**, >97%) were purchased from *TCI*. Poly(ethylene glycol) monomethyl ether methacrylate (n≈9, average M_n = 500, **OEOMA$_{500}$**) and poly(ethylene glycol) monomethyl ether methacrylate (n ≈ 4, average M_n = 276, **OEOMA$_{276}$**), 2-[2-(2-Methoxyethoxy)ethoxy]ethyl acrylate (**MEA**, 90%) and *N,N*-dimethylacrylamide (**DMAA**, >99%), were purchased from *TCI* and passed through a column of basic alumina to remove inhibitor prior to use. Copper(II) bromide ($CuBr_2$, 99%) was purchased from *Thermo Scientific.* 1,4-bis(2-isocyanopropyl)piperazine (**QA**) was purchased from *Strem Chemicals.* 4-Cyano-4-(thiobenzoylthio)pentanoic acid (**CPADB**, 98%) and 2-(dodecylthiocarbonothioylthio)-2-methylpropanoic acid (**DDMAT**, 97%) were purchased from *BLD Pharm.* 2,2'-Azobis[2-(2-imidazolin-2-yl)propane]dihydrochloride (**VA-044**, 95%) was purchased from *Fluorochem.* 2,2′-Azobis(2-methylpropionamidine) dihydrochloride (**AAPH**, 97%) was purchased from *Sigma Aldrich.* 2,2'-Azobis[2-methyl-*N*-(2-hydroxyethyl)propionamide] (**VA-086**, 98%) was purchased from *Wako Pure Chemical Industries.* Water (HPLC grade) was purchased from *Fischer Chemicals.* Dimethyl sulfoxide (DMSO, ≥99.7%) was purchased from *POCH.* Deuterium oxide (99.9%) was purchased from *Deutero.* Tetrachloroauric acid (**$HAuCl_4$** ≥ 99%), hexadecyltrimethylammonium chloride (**CTAC**, 25 wt % in water), hexadecyltrimethylammonium bromide (**CTAB**, ≥99%), sodium borohydride (**$NaBH_4$**), silver nitrate (**$AgNO_3$** ≥99%), sodium hydroxide (98.8%), and 8-hydroxyquinoline (**8-HQL** ≥99%), were purchased from Sigma-Aldrich. Milli-Q water was used in all experiments.

Instrumentation

Nuclear Magnetic Resonance (NMR)

^{1}H NMR spectra were recorded on a Bruker Avance III HD 300 MHz spectrometer with D_2O used as the solvent.

Size Exclusion Chromatography (SEC)

SEC measurements of the synthesized polymers were performed using a Shimadzu (Kyoto, Japan) modular system. The instrument was equipped with CBM-40lite controller, DGU-403 degassing unit, SIL-40 automatic injector, and RID-20A differential refractive-index detector. Separation was performed using a set of three Agilent GRAM columns (two with 3000 Å pores

and one with 100 Å pores), maintained at 40°C. *N,N*-dimethylformamide (HPLC grade) was used as the eluent at a constant flow rate of 1 mL/min. Linear poly(methyl methacrylate) standards were used for calibration.

Transmission Electron Microscopy (TEM)

TEM imaging was performed at the Laboratory of Electron Microscopy, which serves as an imaging core facility at the Nencki Institute of Experimental Biology and is part of the infrastructure of the Polish Euro-BioImaging Node. The Polish Node is supported by the project co-financed by the Minister of Education and Science based on contract No 2022/WK/05 (Polish Euro-BioImaging Node "Advanced Light Microscopy Node Poland").

Ultraviolet–Visible-Near Infrared Spectrophotometry (UV-Vis-NIR)

UV-Vis-NIR measurements were performed using GENESYS 50 UV-Vis spectrophotometer (*Thermo Fisher Scientific*, Waltham, MA, USA).

Image Analysis

To obtain the values of length, width, volume and surface area of nanoparticles, we analyzed Transmission Electron Microscopy (TEM) micrographs using Fiji software equipped with the Biomedgroup & ParticleSizer plugin. The analysis was performed under the following settings: single particle mode was selected, with segmentation parameters set to a circular window radius of 15 px, rolling ball radius of 50 px, and a minimum OTB intensity difference of 16 (8 bit). Shape constraints included a minimum particle area of 700 px^2, minimal convexity of 0.85, and minimal solidity of 0.8, ensuring robust filtering of detected nanoparticles. Additionally, a smoothing factor of 1 was applied. These settings allowed for accurate particle boundary detection and morphological parameter extraction.

Temperature Measurements

Temperature was monitored using an Omega CN9000A temperature controller (Omega Engineering, USA) connected to a type-K thermocouple immersed in the reaction mixture at approximately 3 mm above the bottom of the reaction vessel.

Polymerizations

Polymerizations were carried out in open-to-air 96-well plate or closed, 1.5 mL transparent glass vials. The reaction mixtures were irradiated from below using a *Thorlabs* LED (780 nm, 0.9 W/cm^2 ; catalog number M780LP1).

Supplementary Information

Procedures

Gold Nanobipyramids (NBPs) Synthesis (Figure 2)

NBPs used in our system were synthesized via a modified seed-mediated growth method, as previously described by Chateau et al.[1] Initially, overgrown bipyramidal-like structures were synthesized and employed as seeds for subsequent growth.

The synthesis was carried out using a two-vial system: one containing the growth solution and the other the seed solution. In the first vial, 0.5 mL of 100 mM $HAuCl_4$ solution was mixed with 3 mL of 140 mM cetyltrimethylammonium bromide (CTAB), followed by the addition of 90 µL of 0.8 M tetramethylguanidine (TMG) aqueous solution. After 1–2 min of stirring, 30 µL of 40 mM $AgNO_3$ was added (varied between 12/20/28 µL depending on the targeted position of the absorption band, Figure S1 and Table S1). Higher $AgNO_3$ volumes resulted in more red-shifted LSPR peaks, with approximately 800 nm achieved for 30 µL. The mixture was then reduced by 325 µL of 0.8 M catechol solution (prepared in 25:75 EtOH/H_2O) and subsequently transferred via syringe pump over 3 min into the second vial. The second vial contained either 320 µL or 640 µL of 20 mM seed solution mixed with 1.5 mL of 140 mM CTAB and 13 µL of 40 mM $AgNO_3$, maintained in an oil bath at 55–60 °C. After combining the contents of vial one and two, the solution was stirred for an additional 15 min. The resulting NBPs were purified by centrifugation at 3421 × g for at least 10 min, with the addition of 150 µL of ethanol to reduce solution viscosity. The nanoparticles were redispersed in 1 mM CTAB and subjected to three additional centrifugation cycles. After each centrifugation, the NBPs were redispersed in 20 mM cetyltrimethylammonium chloride (CTAC) solution to facilitate the exchange of the surfactant counterion from bromide (Br^-) to chloride (Cl^-), yielding a final Au^0 concentration of approximately 18 mM.

[1] D. Chateau, A. Desert, F. Lerouge, G. Landaburu, S. Santucci and S. Parola, *ACS Appl. Mater. Interfaces*, **2019**, 11, 39068-39076.

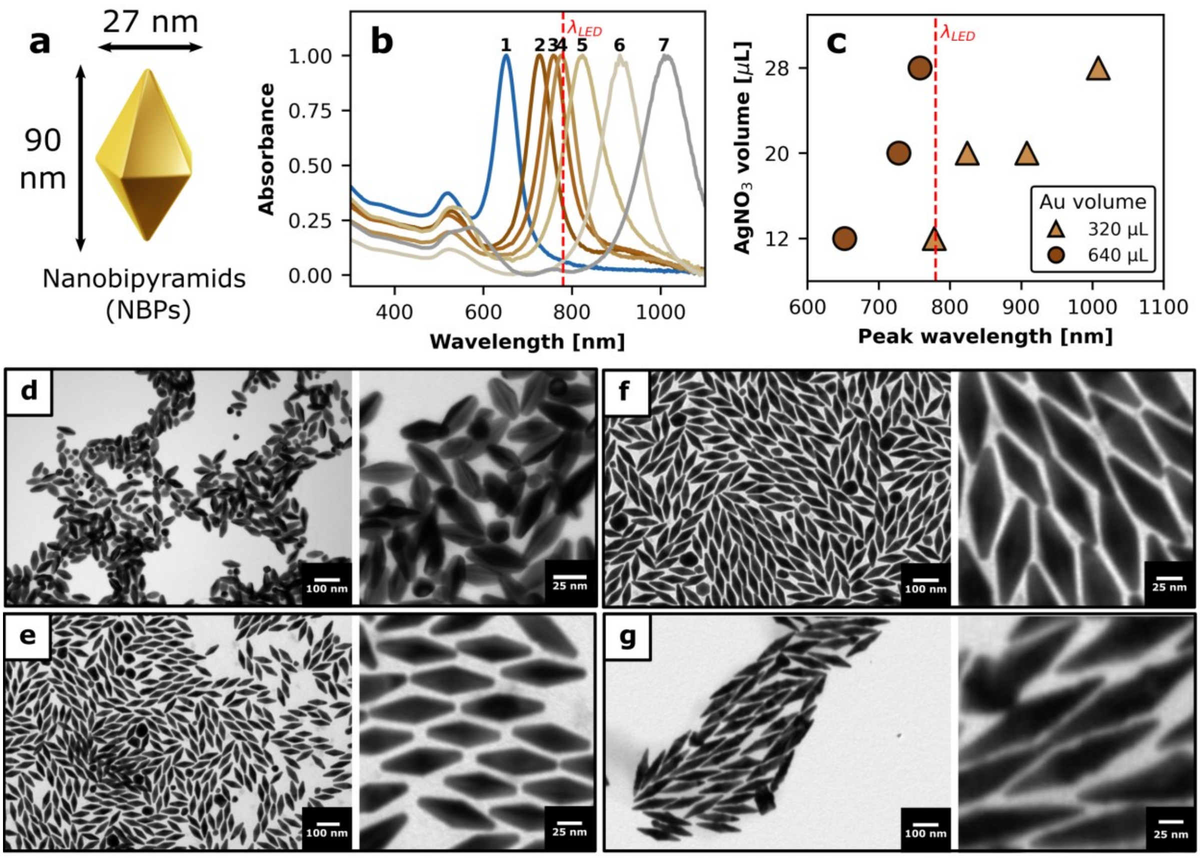

Figure S1. Optical and morphological characterization of NBPs synthesized under varying growth conditions. (d–g) TEM images of bipyramids with progressively increasing size and aspect ratio (AR), tuned by varying $AgNO_3$ concentration and seed quantity. The left panels show large-area views, while the right panels present higher magnification images highlighting shape. (a–c) Normalized UV–Vis–NIR extinction spectra of the samples, demonstrating systematic red-shifts of the longitudinal surface plasmon resonance (LSPR) peak with increasing particle size and AR.

Table S1. Summary of growth conditions and structural/optical properties of NBPs synthesized under varying $AgNO_3$ and seed concentrations.

Symbol	[$AgNO_3$] μl	[Au^0] μl	[Au^{+3}]/[Au^0]	LSPR [nm]	Length [nm]	Width [nm]	Aspect Ratio
1/d		overgrown seeds		652	35	15	2.3
2	12	640	3.91	728	-	-	-
3	20	640	3.91	758	-	-	-
4	28	640	3.91	778	90	27	3.3
5/e	12	320	7.81	824	82	24	3.5
6/f	20	320	7.81	908	112	29	3.8
7/g	28	320	7.81	1008	160	34	4.8

Gold Nanorods (NRs) Synthesis.

Gold nanorods (NRs) were synthesized via a classic seed-mediated growth protocol. Firstly, seed solution preparation. A CTAB solution (100 mM, 4.7 mL) was combined with $HAuCl_4$ (50

mM, 25 µL). Freshly prepared ice-cold $NaBH_4$ (10 mM, 300 µL) was then rapidly injected under vigorous stirring (~1400 rpm). The seed solution was aged for 2 h at 30 °C without disturbance prior to use. Nanorod growth. In a separate vial, CTAB (100 mM, 10 mL) and $HAuCl_4$ (50 mM, 100 µL) were mixed and equilibrated at 30 °C. In selected syntheses, HCl (1 M, 190 µL) was added. After 5 min of gentle stirring, $AgNO_3$ (10 mM, 120 µL) and ascorbic acid (100 mM, 100 µL) were added sequentially, rendering the solution colourless. The seed solution (24 µL) was then injected under vigorous stirring (~1000 rpm) for 10 s, and the mixture was left undisturbed at 30 °C for 2h. To tune the LSPR, the synthesis was repeated using varied volumes of HCl (0–190 µL), $AgNO_3$ (60–120 µL), and seed solution (16–20 µL). The dispersions were purified by centrifugation at 6000 × g for 10 min, followed by redispersion of the precipitate in 20 mM CTAC.

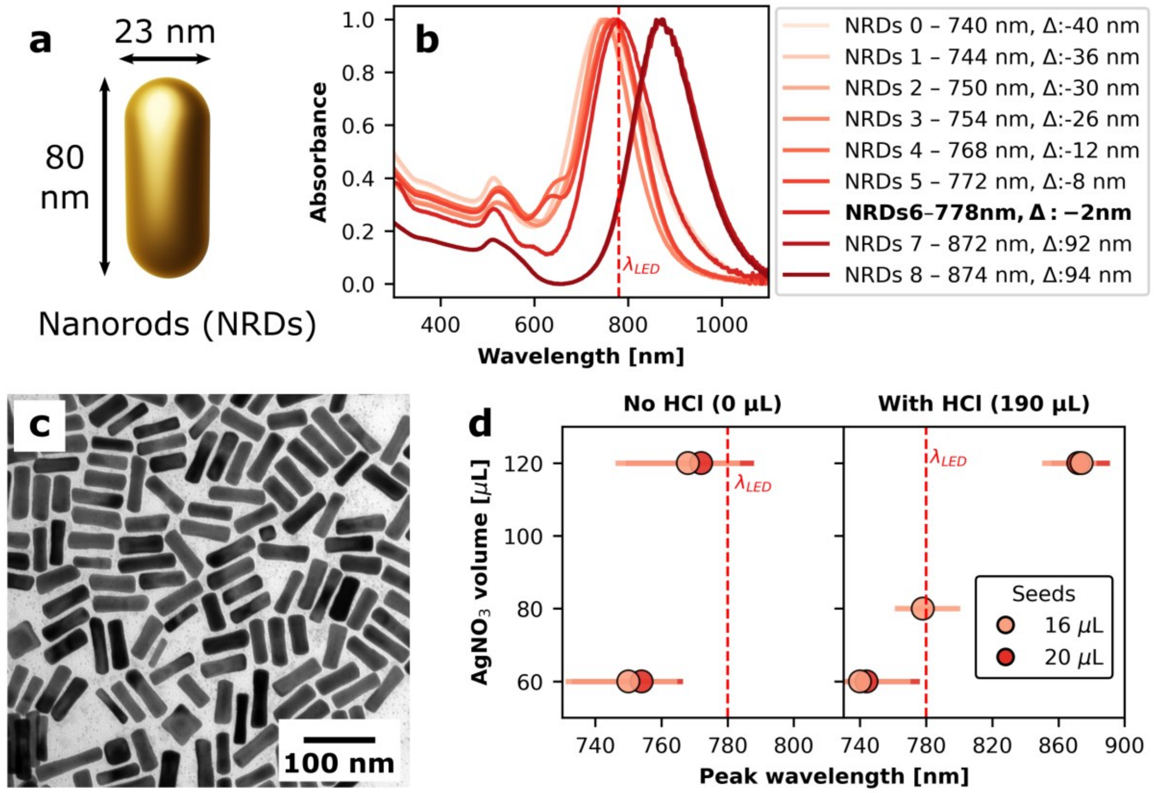

Figure S2. Optical and morphological characterization of gold nanorods (NRs). (a) Schematic representation of the average rod dimensions. (b) Normalized UV–Vis–NIR extinction spectra of NRs showing a systematic red-shift of the longitudinal surface plasmon resonance (LSPR) with increasing $AgNO_3$ and seed concentrations. (c) Representative TEM image of NRs. (d) Correlation between peak wavelength and $AgNO_3$ volume for syntheses performed with and without HCl, highlighting the tunability of aspect ratio and LSPR position. Horizontal bars indicate 95% of the absorption intensity around the peak.

Gold Nanostars (NST) Synthesis.

Seed preparation. Citrate-stabilized gold seeds were prepared via a reverse Turkevich method. An aqueous solution of sodium citrate (2.2 mM, 150 mL) was brought to reflux under vigorous

stirring. A hot solution of $HAuCl_4$ (10 mM, 2.5 mL) was then rapidly injected, resulting in an immediate colour change to deep red. The mixture was maintained at boiling temperature for 15 min, followed by an additional 15 min of stirring during cooling to room temperature. Nanostar growth. A growth solution was prepared by combining $HAuCl_4$ (0.25 mM, 10 mL) with HCl (1 M, 10 µL), after which the seed solution (100 µL, OD ≈ 2.8) was added under gentle stirring. $AgNO_3$ (2–3 mM, 100 µL) and ascorbic acid (100 mM, 50 µL) were subsequently introduced in rapid succession at ~700 rpm. Within 30 s, the solution developed a deep blue or dark green colour, indicative of branched nanostar formation. Synthesis was repeated using varied volumes of $AgNO_3$ (75–400 µL), HCl (10–40 µL), and seed solution (218–436 µL).

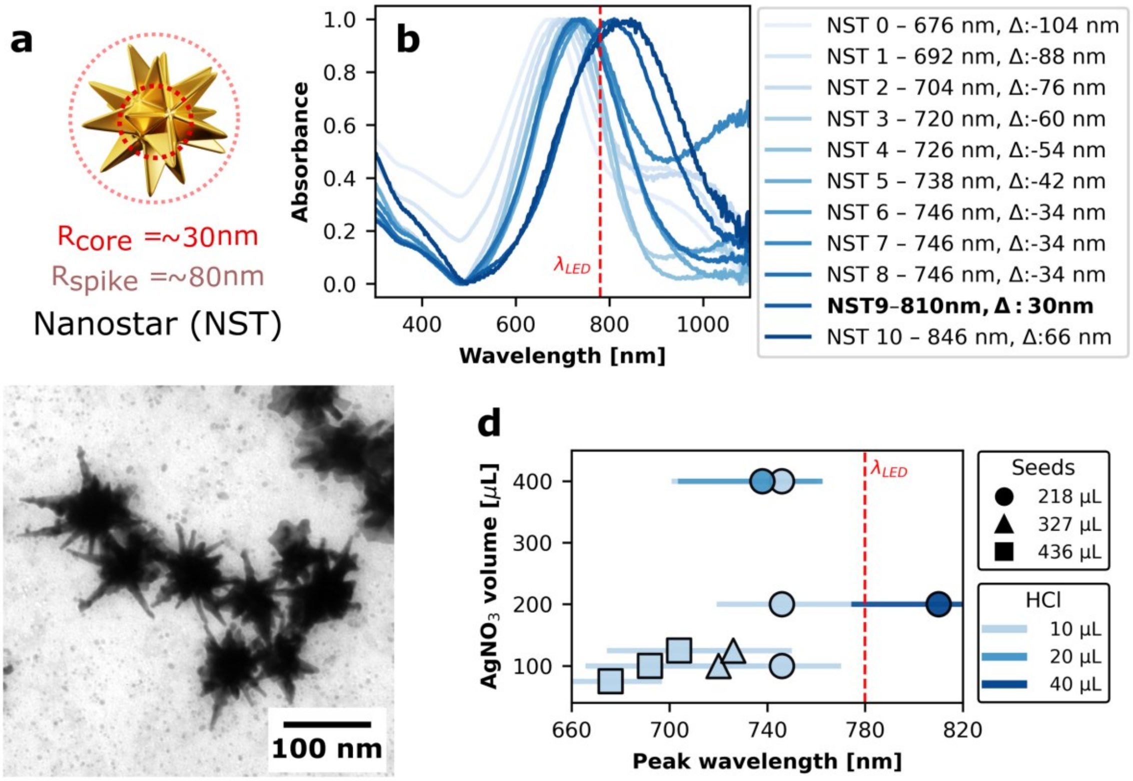

Figure S3. Optical and morphological characterization of gold nanostars (NSTs). (a) Schematic representation of a nanostar with core (~30 nm) and spike (~80 nm) dimensions. (b) Normalized UV–Vis–NIR extinction spectra of NSTs. (c) Representative TEM image of NSTs. (d) Correlation between peak wavelength and $AgNO_3$ volume under varying HCl and seed concentrations, demonstrating tunable plasmonic response near the LED excitation wavelength. Horizontal bars indicate 95% of the absorption intensity around the peak.

Photothermal Conversion Efficiency Measurements (Figure 3)

Prior to polymerization, stock solutions of the following reagents were prepared: $OEOMA_{500}$ (1M in water), HOBiB (15.8 mg in 1.0 mL DMSO), $CuBr_2$ (33.5 mg in 20.0 mL H_2O), TPMA (26.1 mg in 2.0 mL DMSO), AAPH (50.9 mg in 5 mL H_2O), NBPs (18.75 mM of $[Au]^0$ in 10 mM CTAC) and CTAC (100 mM solution in water). The ATRP reaction mixture (1.0 mL total volume) was

prepared in a 1.5 mL transparent glass vial by sequentially adding the following stock solutions: $OEOMA_{500}$ (300 μL), water (382.5 μL), CTAC (200 μL), $CuBr_2$ (60 μL), TPMA (20 μL), HOBiB (20 μL), AAPH (7.5 μL), and NBPs (10 μL). The mixture was vortexed to ensure homogeneity. The resulting final concentrations were: [$OEOMA_{500}$] = 300 mM, [HOBiB] = 1.5 mM, [$CuBr_2$] = 0.45 mM, [TPMA] = 0.9 mM, [AAPH] = 0.28 mM, [CTAC] = 20 mM, and [Au^0] = 0.188 mM. The open vial was then irradiated from below using an LED (780 nm, 0.9 W/cm^2) without stirring. A temperature probe immersed in the solution (Figure S4) recorded the temperature for 1 h during irradiation and for an additional 30 min after the light was turned off. As a control, an identical experiment was conducted without the NBPs.

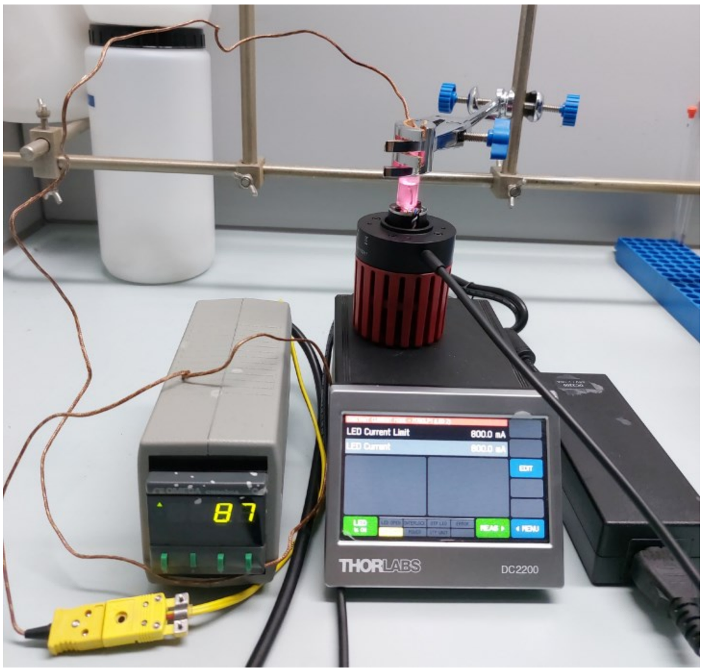

Figure S4. Photothermal performance of NBPs – illustration of temperature measurements.

Heat-Transfer Analysis (Figure 3b)

Here, we combined Wang's transient heat-transfer model with a measured irradiance of E = 0.9 W cm^{-2} at the vial surface to evaluate the net photothermal efficiency η_{net}, approach presented in work of Grzelczak et al.[2].

Fitting Procedure. Temperature–time traces were recorded for nanoparticle-containing (NBPs) and control samples under 780 nm irradiation (0–3600 s ON). These were fitted to Wang's exponential heating model (Eq. S1) and the results were compiled in Table S2:

$$T(t) = T_0 + \frac{a}{b}(1 - e^{-bt}) \quad \text{(S1)}$$

Efficiency Equation. Wang's expression for efficiency is (Eq. S2):

$$\eta = \frac{a_{net}C}{P\left(1-10^{-A_\lambda}\right)} \quad \text{(S2)}$$

where the solution heat capacity was calculated as C (4% *v*/*v* DMSO/water mixture) + optional glass bottom heat capacity. Absorbed fraction which is $1 - 10^{-A_\lambda} \approx 0.9994$ was estimated based on UV-Vis spectrum of BPs at 778 nm, with details in Table S1. Power P was calculated from $P = E \cdot A$ with the vial footprint area is $A = 1.075$ cm^2. We obtain:

- Solution only: $\eta_{net} \approx 0.473$
- Solution + 1 mm glass: $\eta_{net} \approx 0.498$

[2] A. Sánchez-Iglesias and M. Grzelczak, *Small*, **2025**, 21, 2407735.

Table S2. Summary of fit results used for photothermal analysis of NBPs under 780 nm irradiation. Listed are fit parameters from Wang's exponential model (a, b, τ), corrected optical inputs (absorbance were rescaled from 0.2 cm path length to vial geometry), thermal parameters (solution and optional glass contributions), and derived irradiances. Values are used in the calculation of photothermal efficiency shown in Figure S5.

Item	Symbol / Value	Notes
Fitted model	$T(t) = T_0 + \left(\frac{a}{b}\right)(1 - e^{-bt})$	Wang's method;<br>heating window 0–3600 s
BPs starting temp. - T_0	25.0 °C	First point
BPs Fitted - a	0.2210 K s^{-1}	Nonlinear fit
BPs Fitted - b	3.623×10^{-3} s^{-1}	τ =276 s
BPs ΔT_∞	61.0 K	a/b
Control starting temp. - T_0	27.0 °C	First point
Control Fitted - a	0.1100 K s^{-1}	Nonlinear fit
Control Fitted - b	3.736×10^{-3} s^{-1}	τ=268 s
Δa	0.1110 K s^{-1}	a_{BPs} - $a_{control}$
Absorbance	A_{780} = 0.693 @ path = 0.2 cm	From spectrum (LSPR 778 nm)
Effective attenuation	ε_{eff} = 3.465 cm^{-1}	ε_{eff}=A/l
Effective vial absorbance	A_{vial} = 3.223	A_{vial} = $\varepsilon_{eff}\cdot$ h<br>(h=V/A (1.0 mL, Ø11.7 mm))
Absorbed fraction ($1\text{-}10^{-A}$)	0.999401	Used in η equation
Solution heat capacity	4.117 J K^{-1}	From composition
Glass bottom heat capacity	0.215 J K^{-1}	1.0 mm bottom, Ø11.7 mm
Irradiated area	1.075 cm^2	From Ø11.7 mm
η_{net} (soln only / glass)	$\eta_{net} \approx 0.473$ - 0.498	---

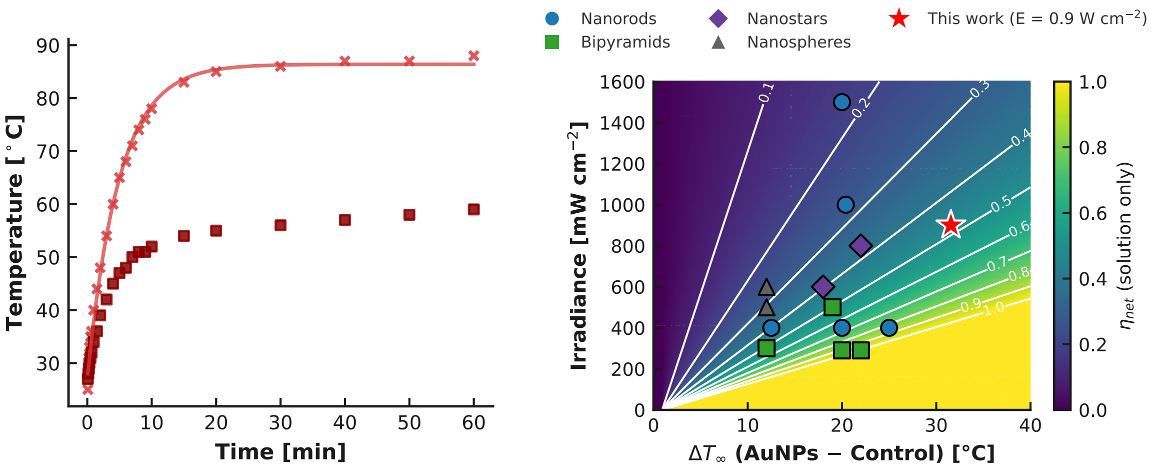

Figure S5. (Left) Experimental heating curves for NBPs (crosses) and control (squares) under 780 nm irradiation, together with fits to Wang's exponential heating model (solid lines). (Right) Heat map showing the relationship between steady-state temperature rise ($\Delta T\infty$, BPs − Control) and irradiance at the vial surface, with color indicating the net photothermal efficiency η_{net}. White contour lines denote $\eta_{net} = 0.1, 0.2, \ldots, 1.0$. The red marks the experimental condition corresponding to the measured LED irradiance of 0.9 $W \cdot cm^{-2}$.

Table S3. Literature values for photothermal heating of gold nanostructures (used in Figures S5-right)

Morphology	Reference	DOI	Data used in plot
**Nanorods (NRs)**	Meyer, S. et al. "Size Effects in Gold Nanorod Light-to-Heat Conversion under Femtosecond Illumination." *J. Phys. Chem. C* **2021**, 125, 21061–21070.	10.1021/acs.jpcc.1c03898	ΔT ~10–25 °C at 400 mW·cm^{-2}; η = 20–37% depending on rod size
	Pedrosa, P. et al. "Selecting High-Performance Gold Nanorods for Photothermal Conversion." *Nanomaterials* **2022**, 12, 4188.	10.3390/nano12234188	ΔT ~20 °C at 1500 mW·cm^{-2}; η ≈ 30–40%
	Zhang, Y. et al. "Improved Photothermal Conversion Efficiency of Gold Nanorods by Optimizing the Aspect Ratio." *Nanoscale Res. Lett.* **2025**, 20, 54.	10.1007/s11051-025-06236-y	ΔT +20.4 °C at 1000 mW·cm^{-2}; η = 36%
**Bipyramids (BPs)**	Focșan, M. et al. "Thermoplasmonic Light-to-Heat Conversion in Colloidal Nanoparticle Solutions and Films." *J. Mater. Chem. C* **2015**, 3, 8445–8452.	10.1039/D5TC01502B	ΔT ~20–22 °C at 290 mW·cm^{-2}; η = 65–74%
	Naik, G. et al. "Size Effect on Photothermal Heating Ability of Gold Bipyramids." *Adv. Optical Mater.* **2025**, 2302041.	10.1002/adom.202501006	ΔT ~18–20 °C at 500 mW·cm^{-2}; η = 60–70%
	Huang, Y. et al. "Plasmonic Photothermal Therapy Using Au Bipyramids: Comparison of Femtosecond and Continuous Wave Laser Irradiation." *Sci. Rep.* **2023**, 13, 1462.	10.1038/s41598-023-30526-x	ΔT ~12 °C at 300 mW·cm^{-2}; η ≈ 30%
**Nanostars (NSTs)**	Khlebtsov, B. et al. "Gold Nanostars and Nanourchins in Biomedicine: Shape, Optical Properties, and Applications." J. Mater. Chem. B 2025, 13, 2341–2360.	10.1039/D4TB01420K	ΔT ~20–25 °C at 500–1000 mW·cm^{-2}; η = 45–55%
	Favi, P. M. et al. "Shape and Surface Chemistry Effects on the Cytotoxicity and Cellular Uptake of Gold Nanoparticles." *J. Biomed. Mater. Res. A* **2015**, 103A, 3449–3462.	10.1002/jbm.a.35518	ΔT ~18 °C at 600 mW·cm^{-2}; η ≈ 50%
**Spheres (SPs)**	Favi, P. M. et al. (same as above).	10.1002/jbm.a.35518	ΔT ~10–15 °C at 500–700 mW·cm^{-2}; η = 20–25%
	Liu, Y. et al. "Gold Nanoparticles for Photothermal Therapy in Tumor Models." *Sci. Rep.* **2017**, 7, 12345.	10.1038/s41598-017-03834-2	ΔT ~12 °C at 400–600 mW·cm^{-2}; η ≈ 20%

Supplementary Information

Extinction Coefficient of NBPs (Figure 2c)

The extinction coefficient of gold nanobipyramids (NBPs) was determined from their UV–Vis–NIR spectrum, calibrated against the elemental gold concentration at 400 nm. Literature calibration established that A (400 nm) = 1.2 in a 1 cm cuvette corresponds to 0.5 mM Au^0 (elemental gold atoms). In our 0.2 cm cuvette, the measured absorbance at 400 nm was A (400 nm) = 0.090, which corresponds to an equivalent 1 cm absorbance of 0.45. From the calibration, this translates to an elemental gold concentration of 1.88×10^{-4} M, or 0.037 $g{\cdot}L^{-1}$.

The average volume of a single NBP was assumed to be 3.5 × 104 nm^3. Using the bulk density of gold (19.32 $g{\cdot}cm^{-3}$), the average mass per nanoparticle was estimated as 6.76×10^{-16} g. Dividing the bulk Au^0 concentration by this per-particle mass yields a particle concentration of 5.47×10^{13} $particles{\cdot}L^{-1}$, equivalent to 9.1×10^{-11} M nanoparticles.

At the localized surface plasmon resonance (LSPR, 778 nm), the measured absorbance was A (778) = 0.383 in a 0.2 cm cuvette. The corresponding path-length–normalized attenuation coefficient is 1.865 cm^{-1}.

From Beer–Lambert's law, the extinction coefficient normalized per mole of nanoparticles is:

$$\varepsilon_{\mathrm{NP}} = \frac{A}{c_{\mathrm{NP}} \cdot l} = 2.05 \times 10^{10}\,\mathrm{M}^{-1}\,\mathrm{cm}^{-1} \quad \text{(S3)}$$

These values are consistent with literature reports of strongly plasmonic Au nanorods and bipyramids, which typically exhibit per-particle extinction coefficients in the range of 10^9–10^{10} $M^{-1}{\cdot}cm^{-1}$

Supplementary Information

Optimization of Photothermal ICAR ATRP of $OEOMA_{500}$ (Table 1)

Prior to polymerization, all reagent stock solutions were prepared according to the procedure described in the photothermal conversion efficiency measurement section. The ATRP "cocktail" (1.0 mL) was prepared in a 1.5 mL Eppendorf tube by adding the following components: $OEOMA_{500}$ (300 μL) and the required amounts of the stock solutions of CTAC, $CuBr_2$, TPMA, HOBiB, AAPH and NBPs. DMSO was added as needed to maintain a constant concentration of 4% (*v*/*v*), and water was added to reach the final 1.0 mL volume. The mixture was vortexed, and a 250 μL aliquot was transferred to a well in a 96-well plate. The plate was athen irradiated from below for 30 min using an LED (780 nm, 0.9 W/cm^2). After the polymerization, samples were collected and analyzed using ^1H NMR and SEC techniques.

Table S4. Optimization studies

Entry	$[Au^0]$ (μM)	$[CuBr_2]$ (equiv)	[TPMA] (equiv)	[AAPH] (equiv)	[CTAC] (mM)	[b]Conv. (%)	[c]$M_{n,th}$	[d]$M_{n,abs}$	[e]$M_{n,app}$	[e]$Đ$	[f]I_{eff} (%)
**1a**	375	0.2	0.4	1.0	20	91	91 200	56 800	47 000	1.37	161
**1b**						93	93 200	56 600	46 800	1.39	165
**1c**						89	89 200	56 000	46 400	1.42	160
**2a**	188	0.2	0.4	1.0	20	92	92 200	58 400	48 100	1.29	158
**2b**						84	84 200	60 700	49 600	1.25	139
**2c**						92	92 200	56 300	46 700	1.32	164
**3a**	94	0.2	0.4	1.0	20	92	92 200	63 000	51 200	1.26	146
**3b**						88	88 200	63 600	51 600	1.25	139
**3c**						95	95 200	62 200	50 600	1.28	153
**3d**						94	94 200	64 600	52 300	1.24	146
**4a**	94	0.2	0.4	0.5	20	73	73 200	60 200	49 600	1.23	122
**4b**						78	78 200	61 500	50 200	1.26	127
**4c**						57	57 200	53 500	44 700	1.23	107
**5a**	94	0.2	0.4	0.25	20	62	62 200	57 700	47 600	1.22	108
**5b**						72	72 200	61 800	50 400	1.25	117
**5c**						63	63 200	56 200	46 600	1.22	112
**5d**						57	57 200	53 900	45 000	1.23	106
**6a**	94	0.2	0.4	0.19	20	58	58 200	57 800	47 600	1.26	100
**6b**						58	58 200	59 100	48 600	1.30	98
**6c**						58	58 200	61 800	50 400	1.27	94
**6d**						58	58 200	60 000	49 100	1.27	97
**7a**	94	0.3	0.6	0.19	20	50	50 200	51 800	43 500	1.23	97
**7b**						56	56 200	56 700	46 900	1.23	99
**7c**						34	34 200	39 600	34 900	1.17	86
**8a**	188	0.3	0.6	0.19	20	60	60 200	59 200	48 600	1.21	102
**8b**						59	59 200	58 000	47 800	1.22	102
**8c**						60	60 200	58 100	47 800	1.25	104
**9a**[g]	94	0.3	0.6	0.19	20	45	45 200	46 500	39 800	1.24	97
**9b**[g]						43	43 200	41 700	36 400	1.25	104
**9c**[g]						52	52 200	50 000	42 300	1.23	104
**10a**[h]	94	0.3	0.6	0.19	20	0	–	–	–	–	–
**11a**	94	0.2	0.4	0.25	40	29	29 200	31 500	28 900	1.24	93
**11b**						14	14 200	19 400	19 300	1.23	73
**12a**	94	0.2	0.4	0.25	30	39	39 200	39 500	34 800	1.20	99
**12b**						34	34 200	34 600	31 200	1.23	99
**12c**						41	41 200	42 800	37 200	1.23	96
**13a**	94	0.2	0.4	0.25	25	52	52 200	51 100	43 000	1.22	102
**13b**						56	56 200	54 000	44 800	1.21	104
**13c**						49	49 200	51 000	42 600	1.20	96
**14a**	94	0.2	0.4	0.25	22.5	49	49 200	47 100	40 300	1.23	104
**14b**						55	55 200	51 800	43 500	1.22	107
**14c**						42	42 200	43 800	37 900	1.22	96
**15a**	94	0.2	0.4	0.25	17.5	50	50 200	48 000	40 900	1.25	105
**15b**						43	43 200	45 600	39 200	1.24	95
**15c**						56	56 200	52 300	43 900	1.25	107

[a)]Reaction conditions: $[OEOMA_{500}]/[HOBiB]/[CuBr_2]/[TPMA]/[AAPH]$ = 200/1/x/x/x, $[Au^0]$ = 94–375 μM, $[OEOMA_{500}]$ = 300 mM, in water with [CTAC] = 20 mM and DMSO (4% *v*/*v*) in an open-air 96-well plate at a volume of 250 μL, irradiated for 30 min under NIR LED (780 nm, 0.9 W/cm^2). [b)]Determined by ^{1}H NMR spectroscopy. [c)]$M_{n,th}$ = 200 × conv. × $MW_{OEOMA500}$ + MW_{HOBIB}. [c)] Determined by Mark–Houwink calibration. [d)]Determined by SEC analysis (DMF as eluent) calibrated with poly(methyl methacrylate) standards. [f)]Initiation efficiency (I_{eff}) = $M_{n,th}/M_{n,abs}$. [g)]VA-044 instead of AAPH; [h)]VA-086 instead of AAPH, 1.5 h of NIR irradiation.

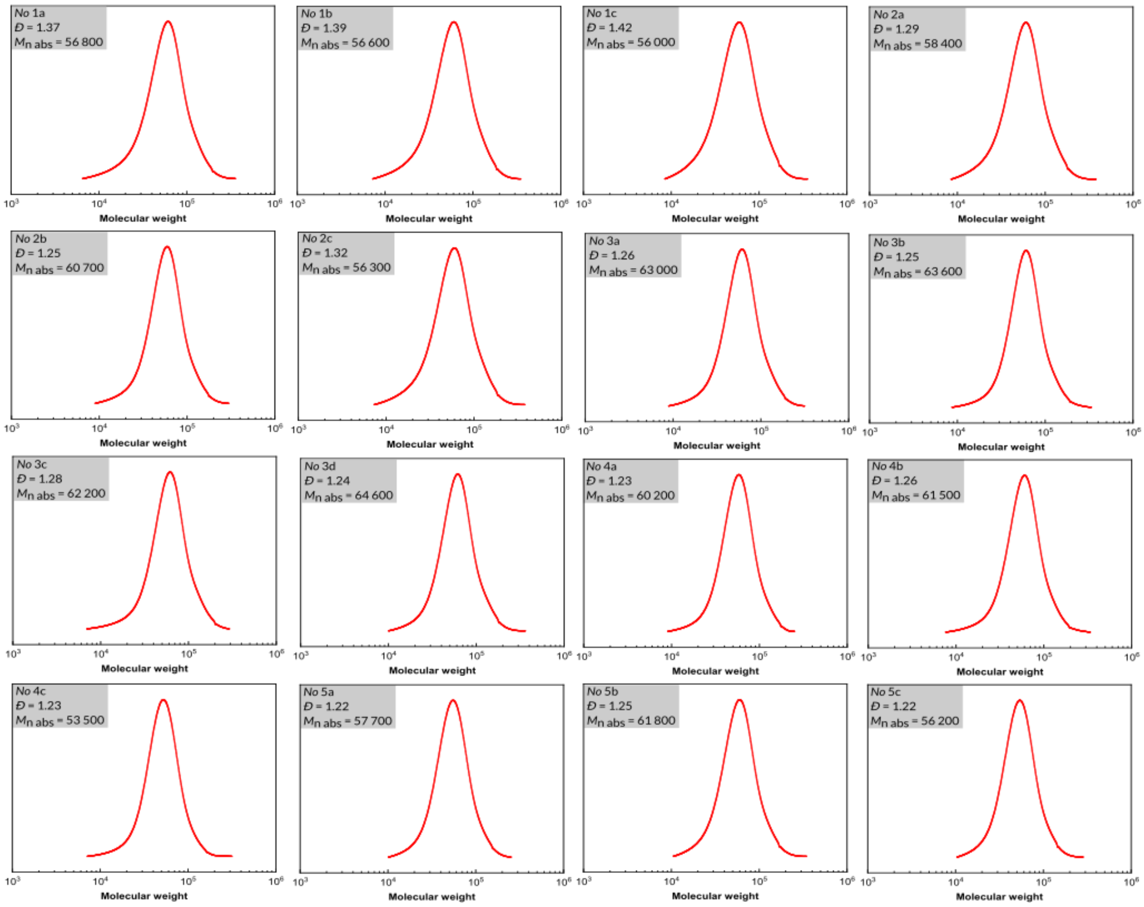
No 1a
Đ = 1.37
Mn abs = 56 800
No 1b
Đ = 1.39
Mn abs = 56 600
No 1c
Đ = 1.42
Mn abs = 56 000
No 2a
Đ = 1.29
Mn abs = 58 400
No 2b
Đ = 1.25
Mn abs = 60 700
No 2c
Đ = 1.32
Mn abs = 56 300
No 3a
Đ = 1.26
Mn abs = 63 000
No 3b
Đ = 1.25
Mn abs = 63 600
No 3c
Đ = 1.28
Mn abs = 62 200
No 3d
Đ = 1.24
Mn abs = 64 600
No 4a
Đ = 1.23
Mn abs = 60 200
No 4b
Đ = 1.26
Mn abs = 61 500
No 4c
Đ = 1.23
Mn abs = 53 500
No 5a
Đ = 1.22
Mn abs = 57 700
No 5b
Đ = 1.25
Mn abs = 61 800
No 5c
Đ = 1.22
Mn abs = 56 200
Molecular weight

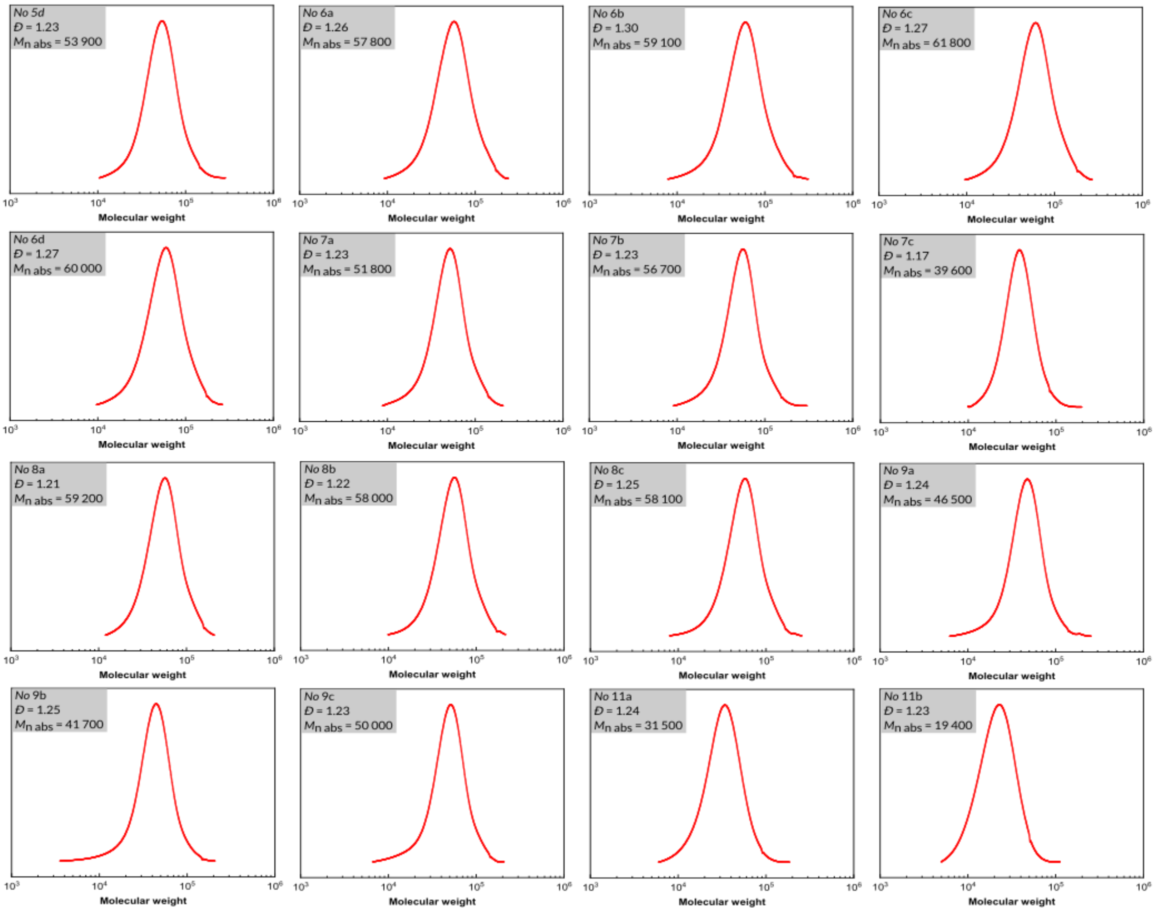
No 5d
Đ = 1.23
Mn abs = 53 900
No 6a
Đ = 1.26
Mn abs = 57 800
No 6b
Đ = 1.30
Mn abs = 59 100
No 6c
Đ = 1.27
Mn abs = 61 800
No 6d
Đ = 1.27
Mn abs = 60 000
No 7a
Đ = 1.23
Mn abs = 51 800
No 7b
Đ = 1.23
Mn abs = 56 700
No 7c
Đ = 1.17
Mn abs = 39 600
No 8a
Đ = 1.21
Mn abs = 59 200
No 8b
Đ = 1.22
Mn abs = 58 000
No 8c
Đ = 1.25
Mn abs = 58 100
No 9a
Đ = 1.24
Mn abs = 46 500
No 9b
Đ = 1.25
Mn abs = 41 700
No 9c
Đ = 1.23
Mn abs = 50 000
No 11a
Đ = 1.24
Mn abs = 31 500
No 11b
Đ = 1.23
Mn abs = 19 400
Molecular weight

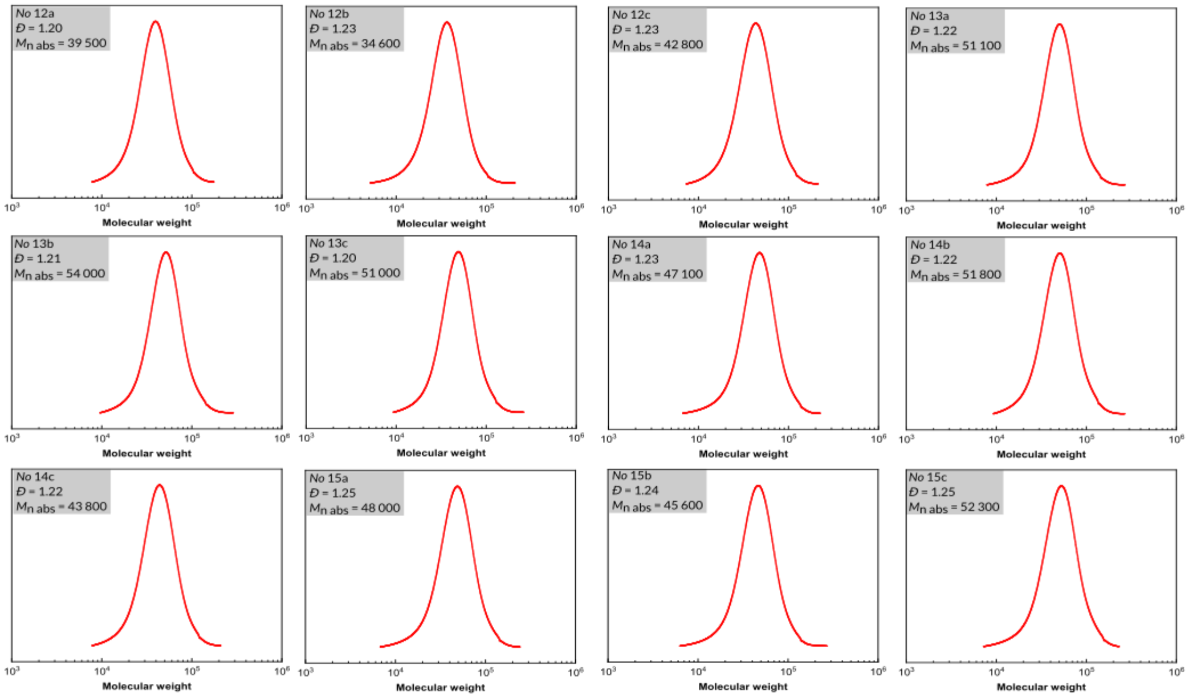

Figure S6. SEC traces for the polymerizations from Table 1.

Control Experiments of Photothermal ICAR ATRP (Table 2)

Prior to polymerization, all reagent stock solutions were prepared according to the procedure described in the *Photothermal Conversion Efficiency Measurements* section. The ATRP "cocktail" (1.0 mL) was prepared in a 1.5 mL transparent glass vial by sequentially adding the following stock solutions: $OEOMA_{500}$ (300 µL) and optionally CTAC (200 µL), HOBiB (20 µL), TPMA (20 µL), $CuBr_2$ (60 µL), AAPH (7.5 µL), and an appropriate volume of NPs to reach a final Au^0 concentration in the polymerization mixture of 188 µM (0.383 ABS at 780 nm for 2 mm optical path). DMSO was added as needed to maintain a constant concentration of 4% (*v*/*v*), and water was added to reach a final 1.0 mL volume. The mixture was vortexed. The vial was then either placed above an LED (780 nm, 0.9 W/cm^2) and irradiated for 1 h or placed in a heating block and kept at 88 °C for 1 h. After the polymerization, samples were collected and analyzed using ^{1}H NMR and SEC techniques.

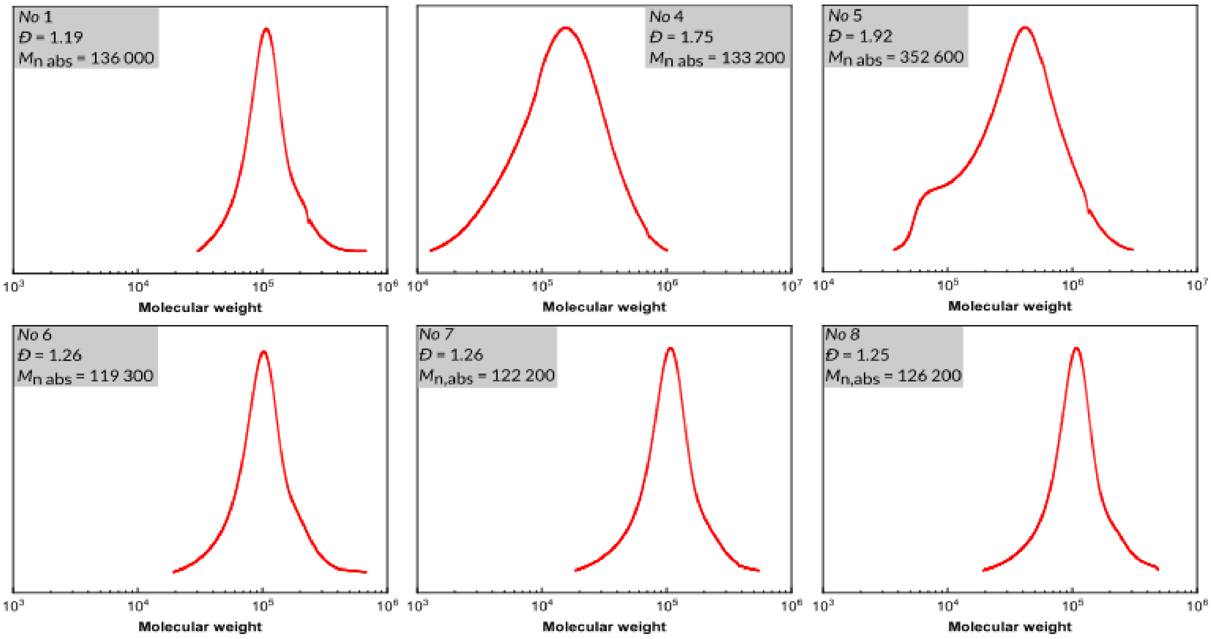

Figure S7. SEC traces for the polymerizations from Table 2.

Colloidal Stability of NPs

Prior to polymerization, all reagent stock solutions were prepared according to the procedure described in the *Photothermal Conversion Efficiency Measurements* section. The ATRP "cocktail" (1.0 mL) was prepared in a 1.5 mL transparent glass vial by sequentially adding the following stock solutions: $OEOMA_{500}$ (300 µL), CTAC (200 µL), HOBiB (20 µL), TPMA (20 µL), $CuBr_2$ (60 µL), AAPH (7.5 µL), and an appropriate volume of NPs to reach a final Au^0 concentration in the polymerization mixture of 188 µM (0.383 ABS at 780 nm for 2 mm optical path). DMSO was added to maintain a constant concentration of 4% (*v*/*v*), and water was added to reach a final 1.0 mL volume. The mixture was vortexed. The vial was placed above an LED (780 nm, 0.9 W/cm^2) and irradiated for 1 h After the polymerization, samples were collected and analyzed using ^{1}H NMR and SEC techniques.

To evaluate the effect of polymerization conditions on the stability and optical properties of plasmonic nanoparticles, UV–Vis–NIR spectra were recorded for the polymerization mixtures before and after the ATRP reaction (Figure S8). "Before" samples were measured within 30 min after mixing NPs with the ATRP cocktail, while "after" samples were collected within 30 min following completion of the ATRP procedure.

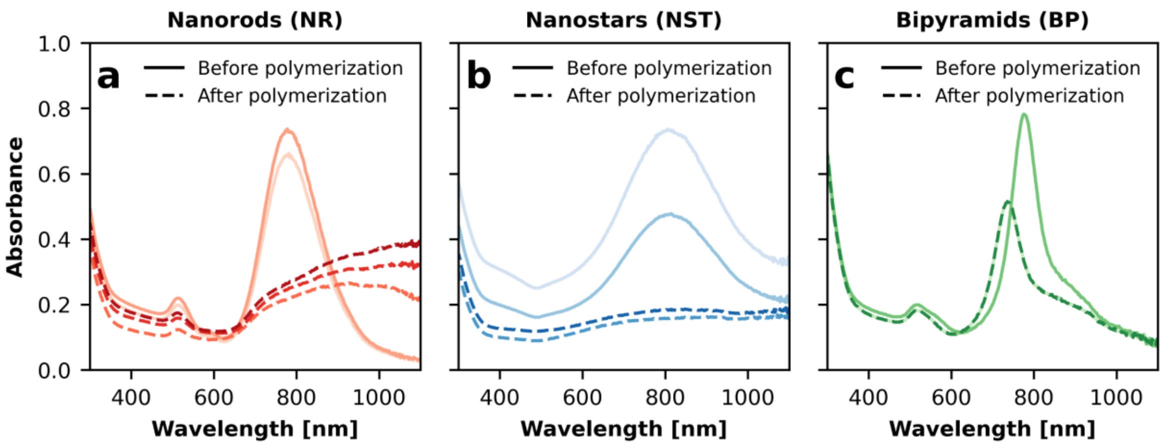

Figure S8. UV–Vis–NIR absorbance spectra of nanorods (NR), nanostars (NST), and nanobipyramids (BPs) before and after NIR-initiated ATRP.

Impact of Nanoparticle Morphology on Photothermal ICAR ATRP.

To evaluate the ICAR ATRP performance as a function of NPs morphology, three distinct plasmonic shapes were synthesized: nanorods (NRs), nanobipyramids (NBPs), and nanostars (NSTs). The syntheses were optimized to achieve comparable optical properties—specifically, a LSPR maximum centered near the 780 nm LED excitation wavelength—and similar nanoparticle volumes. After optimization, NRs sample 6 and NST sample 9 (with optical properties shown in Figure S2b and Figure S3b, respectively) were selected as the closest in spectral response to the NBPs used in this study. ATRP performance was evaluated under two normalization conditions: (i) normalized Au^0 concentration and (ii) normalized absorbance (ABS) at 780 nm. For the equal Au^0 concentration condition (Figure S9a), the total gold content in the polymerization mixture was adjusted to (188 ± 10) µM, based on the calibration ratio of 0.5 mM Au^0 = 1.2 ABS at 400 nm (1 cm optical path). For the equal absorbance condition (Figure S9b), the normalization point was set to (0.727 ± 0.01) ABS at 780 nm (2 mm optical path). As shown in Figure S9a, when samples normalized to equal gold concentration (i.e., similar NP volumes and particle count), NRs and NSTs exhibited lower excitation efficiency at 780 nm. Among the tested morphologies, NBPs displayed the highest optical efficiency, followed by NRs, while NSTs were the least efficient. The reduced performance of NSTs can also be attributed to their lower monodispersity in shape and size, compared to higher purity of NRs and NBPs typically around 95–99%.

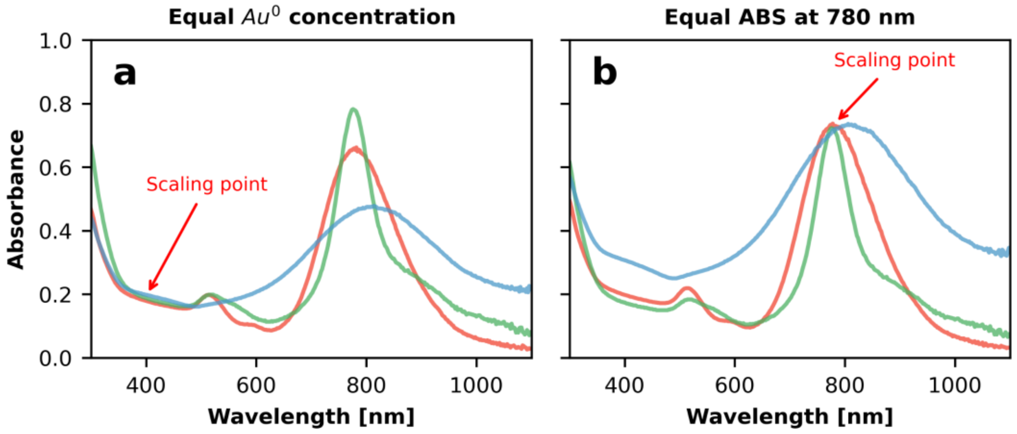

Figure S9. Comparison of absorption spectra of bipyramids (NBPs), nanorods (NRs), and nanostars (NST) under different normalization conditions. (a) Spectra scaled to equal Au^0 concentration. (b) Spectra normalized to equal absorbance at 780 nm. Red arrows indicate the spectral regions used for scaling.

Prior to polymerization, all reagent stock solutions were prepared according to the procedure described in the *Photothermal Conversion Efficiency Measurements* section. The ATRP "cocktail" (1.0 mL) was prepared in a 1.5 mL transparent glass vial by sequentially adding the following stock solutions: $OEOMA_{500}$ (300 µL), CTAC (200 µL), HOBiB (20 µL), TPMA (20 µL), $CuBr_2$ (60 µL), AAPH (7.5 µL), and an appropriate volume of NPs. DMSO was added to maintain a constant concentration of 4% (*v*/*v*), and water was added to reach a final 1.0 mL volume. The mixture was vortexed. The vial was placed above an LED (780 nm, 0.9 W/cm^2) and irradiated for 1 h After the polymerization, samples were collected and analyzed using ^{1}H NMR and SEC techniques.

Table S5. Photothermal ICAR ATRP with different nanoparticle morphologies.

Entry	NPs	^{b}Conv. (%)	$^cM_{n,th}$	$^dM_{n,abs}$	$^eM_{n,app}$	$^eĐ$	$^fI_{eff}$ (%)
1	NBPs	72	144 200	136 000	96 600	1.19	106
2	NRs, equal [Au^0]	71	142 200	122 200	88 400	1.26	116
3	NRs, equal abs	71	142 200	117 000	85 300	1.30	122
4	NSTs, equal [Au^0]	70	140 200	126 200	90 800	1.25	111
5	NSTs, equal abs	71	142 200	117 000	85 300	1.30	122

$^{a)}$ Reaction conditions: [$OEOMA_{500}$]/[HOBiB]/[$CuBr_2$]/[TPMA]/[AAPH] = 400/1/0.6/1.2/0.38, [$OEOMA_{500}$] = 300 mM, [HOBiB] = 0.75 mM, [$CuBr_2$] = 0.45 mM, [TPMA] = 0.9 mM, [AAPH] = 0.285 mM, [CTAC] = 20 mM. Polymerizations were conducted in an aqueous solutions containing DMSO (4% *v*/*v*) at a scale of 1 mL in sealed vials in the presence of air (non-degassed solutions). NIR LED irradiation (780 nm, 0.9 W/cm^2) was applied for 60 min. $^{b)}$ Determined by ^{1}H NMR spectroscopy. $^{c)}$ $M_{n,th}$ = 400 × conv. × $MW_{OEOMA500}$ + MW_{HOBIB}. $^{d)}$ Determined by Mark–Houwink calibration. $^{e)}$ Determined by SEC analysis (DMF as eluent) calibrated with poly(methyl methacrylate) standards. $^{f)}$ Initiation efficiency (I_{eff}) = $M_{n,th}/M_{n,abs}$.

Kinetics of Photothermal ICAR ATRP of $OEOMA_{500}$ (Figure 4)

All reagent stock solutions were prepared according to the procedure described in the photothermal conversion efficiency measurement section. The HOBiB stock solutions were prepared as follows: for a DP_T of 200, 15.8 mg of HOBiB was dissolved in 1.0 mL of DMSO, for a DP_T of 400, 15.8 mg of HOBiB was dissolved in 2.0 mL of DMSO. The reaction mixture was prepared in a 1.5 mL transparent glass vial by adding the following stock solutions: $OEOMA_{500}$ (450 µL), $CuBr_2$ (90 µL), TPMA (30 µL), HOBiB (30 µL), AAPH (11.3 µL), CTAC (300 µL), NBPs (15 µL), and water (572.8 µL). The vial was sealed with a cap and the mixture was vortexed. The resulting final concentrations were: [$OEOMA_{500}$] = 300 mM, [HOBiB] = 1.5 mM for DP_T = 200 and 0.75 mM for DP_T = 400, [CTAC] = 20 mM, [$CuBr_2$]= 0.45 mM, [TPMA] = 0.9 mM, [AAPH] = 0.28 mM, [DMSO] = 4% (*v*/*v*), and [Au^0] = 0.188 mM (0.383 ABS at 780 nm for 2 mm optical path). The vial was then placed above an LED (780 nm, 0.9 W/cm^2). At indicated time intervals, 150 µL aliquots were withdrawn via a microliter syringe and immediately quenched with 20 µL of a 1,4-bis(3-isocyanopropyl)piperazine solution (10 mg/mL in H_2O). The quenched samples were then analyzed using ^1H NMR and SEC techniques.

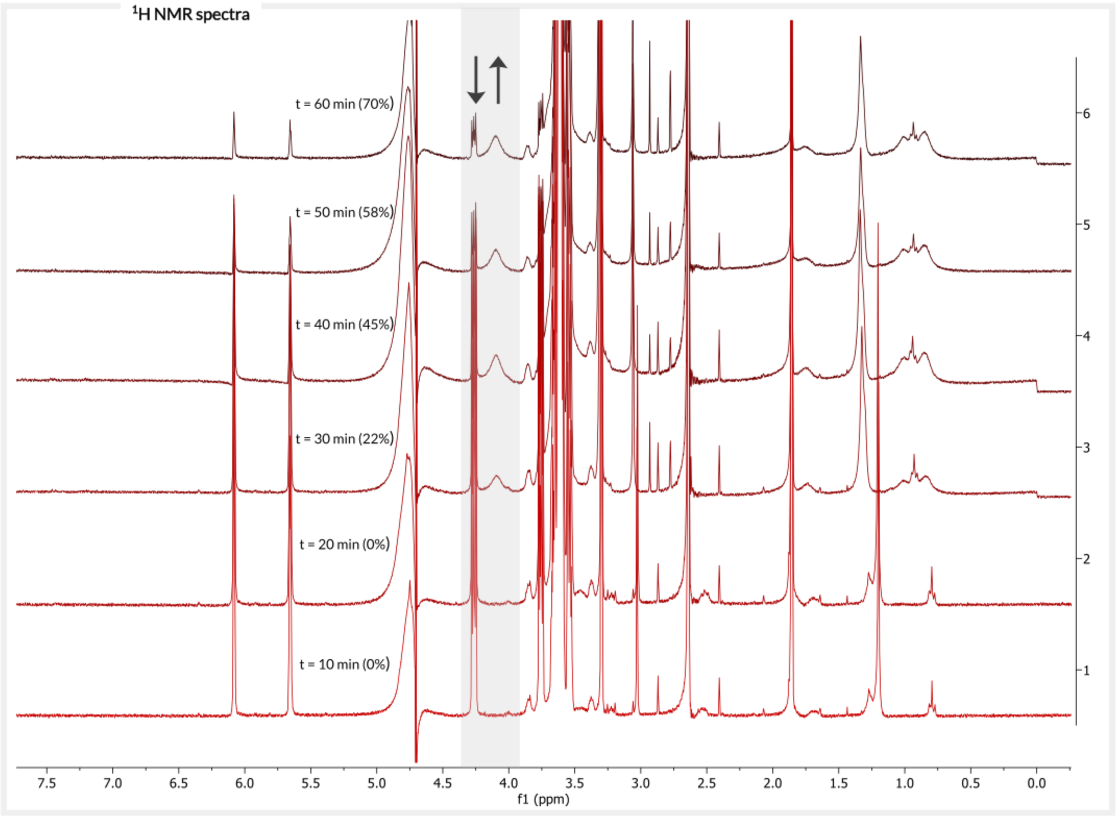

Figure S10. ^1H NMR spectrum of the crude polymerization mixture for DP_T = 200.

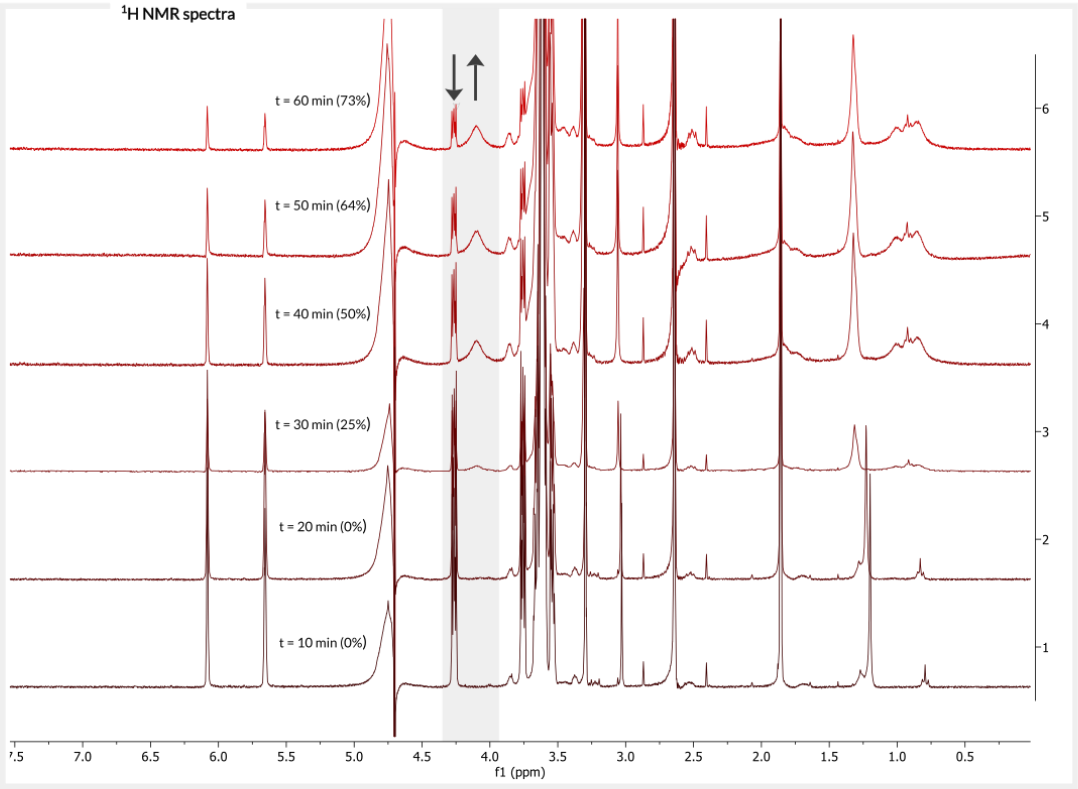

Figure S11. ¹H NMR spectrum of the crude polymerization mixture for DP_T = 400.

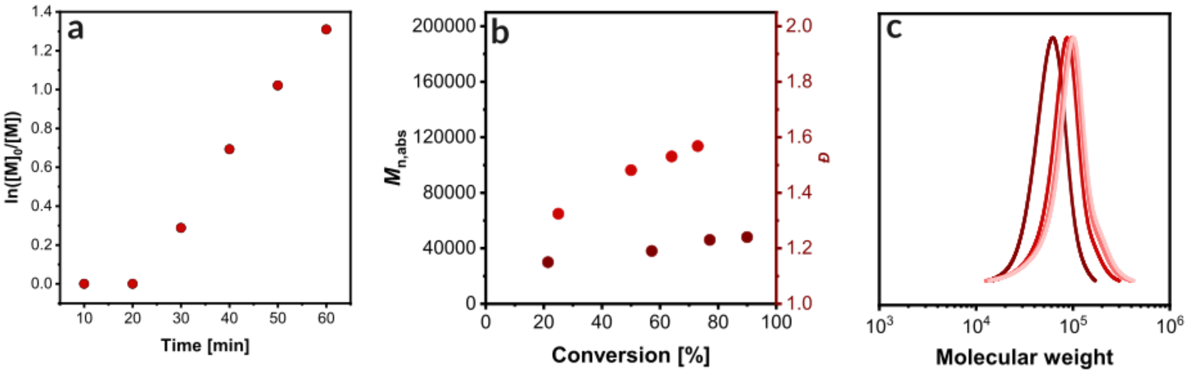

Figure S12. Kinetic studies of polymerization of $OEOMA_{500}$ for DP_T = 400. (a) First-order kinetic plot of $\ln([M]_0/[M])$ versus time. (b) Evolution of number-average molecular weight (M_n) and dispersity ($Đ$) with monomer conversion. (c) Evolution of SEC traces over time.

Temporal Control of Photothermal ICAR ATRP (Figure 5a)

The ATRP reaction mixture was prepared according to the procedure described for the kinetic studies, using a molar ratio of [$OEOMA_{500}$]/[HOBiB] = 200/1. The polymerization mixture was subjected to intermittent irradiation (780 nm, 0.9 W/cm^2), with the light being turned *on* and *off* at 15-minute intervals. At the indicated time, a 150 µL aliquot was withdrawn via a microliter syringe and immediately quenched with 20 µL of a 1,4-bis(3-isocyanopropyl)piperazine solution (10 mg/mL in H_2O). The quenched samples were then analyzed using ¹H NMR and SEC techniques.

Temporal Control of Photothermal ICAR ATRP Under Anaerobic Conditions

The ATRP reaction mixture was prepared according to the procedure described for the kinetic studies, using a molar ratio of [$OEOMA_{500}$]/[HOBiB] = 200/1. The mixture was deoxygenated prior to polymerization, and then subjected to intermittent irradiation (780 nm, 0.9 W/cm^2), with the light being turned *on* and *off* at 15-minute intervals. At the indicated time, a 150 μL aliquot was withdrawn via a microliter syringe and immediately quenched with 20 μL of a 1,4-bis(3-isocyanopropyl)piperazine solution (10 mg/mL in H_2O). The quenched samples were then analyzed using ^{1}H NMR and SEC techniques.

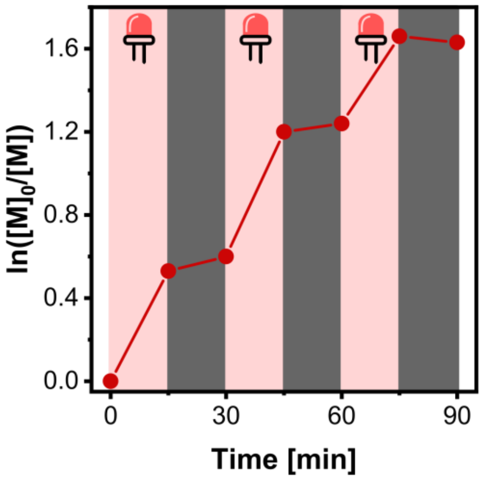

Figure S13. Photothermal ICAR ATRP under anaerobic conditions.

Block Copolymerization (Figure 5b)

Stock solutions of $OEOMA_{500}$, $CuBr_2$, TPMA, AAPH, CTAC and NBPs were prepared according to the procedure described in the *Photothermal Conversion Efficiency measurements* section. A separate stock solution of the initiator, HOBiB (150 mM), was prepared by dissolving 31.6 mg of HOBiB in 1.0 mL of DMSO.

Synthesis of p($OEOMA_{500}$)-*b*-p($OEOMA_{500}$) Diblock Copolymer

The initial ATRP reaction mixture (1.0 mL, DP_T = 100) was prepared in a 1.5 mL transparent glass vial by adding the following stock solutions: $OEOMA_{500}$ (300 μL), HOBiB (20 μL, 150 mM), $CuBr_2$ (60 μL), TPMA (20 μL), AAPH (7.5 μL), CTAC (200 μL), water (380 μL) and NBPs (10 μL). The reaction mixture was vortexed. The resulting final concentrations were: [$OEOMA_{500}$] = 300 mM, [HOBiB] = 3.0 mM, [$CuBr_2$] = 0.45 mM, [TPMA] = 0.9 mM, [AAPH] = 0.28 mM, [Au^0] = 0.188 mM, [CTAC] = 20 mM and [DMSO] (4% v/v). The vial was then placed above an LED (780 nm, 0.9 W/cm^2) and irradiated for 25 min. The macroinitiator p($OEOMA_{500}$) was synthesized with a monomer conversion of 39% ($M_{n,abs}$ = 27 500, $M_{n,app}$ =

25 800, $Đ$ = 1.19). Following the initial polymerization, two samples (200 µL each) were taken from the post-polymerization mixture. The first was analyzed using ^{1}H NMR and SEC techniques. The second sample, containing the crude p($OEOMA_{500}$) macroinitiator, was used without further purification for a chain-extension polymerization to target a second block with a DP_T of 400. The ATRP "cocktail" for a chain-extension polymerization was prepared in a 1.5 mL glass vial by adding: 240 µL of $OEOMA_{500}$ (1M in water), crude p($OEOMA_{500}$) solution (200 µL), CTAC stock (160 µL), water (306 µL), $CuBr_2$ stock (48 µL), TPMA stock (16 µL), AAPH stock (6 µL), DMSO (16 µL) and NBPs (8 µL). The vial was sealed with a cap and vortexed. The vial was then placed above an LED (780 nm, 0.9 W/cm^2, bottom-up) and irradiated for 30 min. After the polymerization, SEC analysis showed a clear shift toward higher molecular weights: $M_{n,abs}$ = 159 100, $M_{n,app}$ = 109 900, and $Đ$ = 1.24.

Synthesis of p($OEOMA_{500}$)-*b*-p($OEOMA_{276}$) Diblock Copolymer

The ATRP "cocktail" (1.0 mL) with a target DP of 100 was prepared the same way as described above. The macroinitiator p($OEOMA_{500}$) was synthesized with 34% monomer conversion ($M_{n,app}$ = 21 500, $Đ$ = 1.20). Two samples (200 µL each) were then taken from the post-polymerization mixture. The first one was analyzed using ^{1}H NMR and SEC techniques. The second sample, containing the crude p($OEOMA_{500}$) macroinitiator, was used without further purification for a chain-extension polymerization to target a second block with a DP_T of 400. The ATRP "cocktail" for a chain-extension polymerization was prepared in a 1.5 mL glass vial by adding 66.2 mg of $OEOMA_{276}$ (61.3 µL), crude p($OEOMA_{500}$) solution (200 µL), CTAC stock (160 µL), water (484.7 µL), $CuBr_2$ stock (48 µL), TPMA stock (16 µL), AAPH stock (6.0 µL), DMSO (16 µL), and NBPs (8 µL). The vial was sealed with a cap and vortexed. The vial was then placed above an LED (780 nm, 0.9 W/cm^2) and irradiated for 30 min. After the

polymerization, SEC analysis showed a clear shift toward higher molecular weights: $M_{n,app}$ = 60 200, and $Đ$ = 1.23.

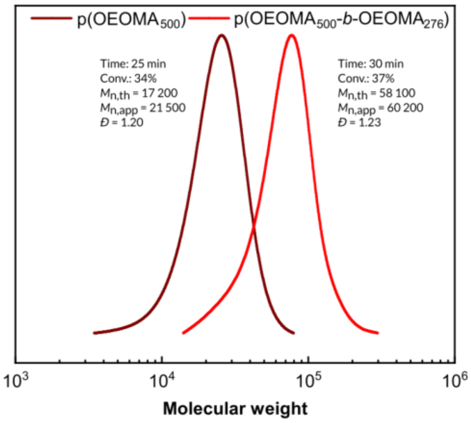

Figure S14. Chain extension of $pOEOMA_{500}$ macroinitiator with $OEOMA_{276}$ monomer.

Photothermal ICAR ATRP of $OEOMA_{500}$ with Varying DP_T (Table 3)

The target degrees of polymerization (DP_T) were varied by adjusting the HOBiB concentration (0.3–6 mM), while the concentrations of $OEOMA_{500}$ (300 mM), CTAC (20 mM), $CuBr_2$ (0.45 mM), TPMA (0.9 mM), AAPH (0.28 mM), DMSO (4% v/v), and NBPs ($[Au^0]$ = 0.188 mM) were fixed for each reaction. HOBiB stock solutions of different concentrations (150, 75, 37.4, 25, 18.7, or 15 mM) were prepared by diluting the HOBiB solution (300 mM, 63.2 mg in 1.0 mL DMSO) with DMSO.

The ATRP "cocktail" (1.0 mL) was prepared in a 1.5 mL transparent glass vial, according to the general procedure by adding HOBiB stock solution (20 μL) at a specific concentration (300, 150, 75, 37.4, 25, 18.7, or 15 mM) to obtain the target DP (DP_T = 50, 100, 200, 400, 600, 800 or 1000, respectively). The vial was closed with a cap and vortexed. The vial was then placed above an LED (780 nm, 0.9 W/cm^2) and irradiated for 30 min. After the polymerization, samples were collected and analyzed using ^{1}H NMR and SEC techniques.

Photothermal RAFT Polymerization of Different Monomers (Table 4)

Prior to polymerization, the following stock solutions were prepared: $OEOMA_{500}$ (1M in water), CPADB (10.5 mg in 1.0 mL DMSO), DDMAT (68.4 mg in 1.0 mL DMSO), AAPH (50.9 mg in 5 mL H_2O), NBPs (18.75 mM in 10 mM CTAC), and CTAC (100 mM solution in water). Three different reaction mixtures (1.0 mL each) were prepared to target a final monomer concentration of 300 mM.

- Entry 1: In a 1.5 mL transparent glass vial, 300 µL of $OEOMA_{500}$ stock solution, 200 µL of CTAC stock solution, 40 µL of CPADB stock solution, 7.5 µL of AAPH stock solution, 10 µL of NBPs stock solution and 442.5 µL of water were added.
- Entry 2: In a 1.5 mL transparent glass vial, 65.4 mg of MEA (61.7 µL) was weighed. Then, 200 µL of CTAC stock solution, 8 µL of DDMAT stock solution, 7.5 µL of AAPH stock solution, 32 µL of DMSO, 680.8 µL of water and 10 µL of NBPs stock solution were added.
- Entry 3: In a 1.5 mL transparent glass vial, 29.7 mg of DMAA (30.9 µL) was weighed. Then, 200 µL of CTAC stock solution, 8 µL of DDMAT stock solution, 7.6 µL of AAPH stock solution, 32 µL of DMSO, 711.5 µL of water, and 10 µL of NBPs stock solution were added.

The mixtures were vortexed, then placed above an LED (780 nm, 0.9 W/cm^2) and irradiated for the specified duration. After the polymerization, samples were collected and analyzed using ^1H NMR and SEC techniques.

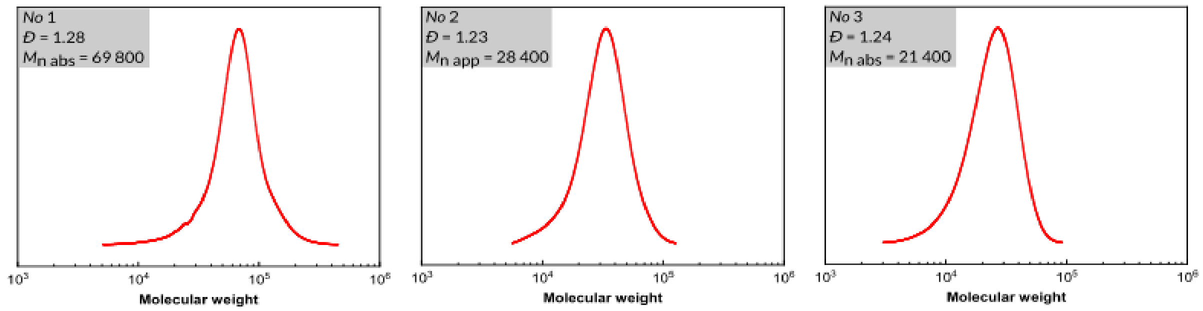

Figure S15. SEC traces for the polymerizations from Table 4.
